# Essential role of M1 macrophages in blocking cytokine storm and pathology associated with murine HSV-1 infection

**DOI:** 10.1371/journal.ppat.1009999

**Published:** 2021-10-15

**Authors:** Ujjaldeep Jaggi, Harry H. Matundan, Jack Yu, Satoshi Hirose, Mathias Mueller, Floyd L. Wormley, Homayon Ghiasi

**Affiliations:** 1 Center for Neurobiology and Vaccine Development, Ophthalmology Research, Department of Surgery, Cedars-Sinai Burns & Allen Research Institute, Los Angeles, California, United States of America; 2 Institute of Animal Breeding and Genetics, University of Veterinary Medicine Vienna, Vienna, Austria; 3 Department of Biology, Texas Christian University, Fort Worth, Texas, United States of America; Oklahoma State Univeristy, UNITED STATES

## Abstract

Ocular HSV-1 infection is a major cause of eye disease and innate and adaptive immunity both play a role in protection and pathology associated with ocular infection. Previously we have shown that M1-type macrophages are the major and earliest infiltrates into the cornea of infected mice. We also showed that HSV-1 infectivity in the presence and absence of M2-macrophages was similar to wild-type (WT) control mice. However, it is not clear whether the absence of M1 macrophages plays a role in protection and disease in HSV-1 infected mice. To explore the role of M1 macrophages in HSV-1 infection, we used mice lacking M1 activation (M1^-/-^ mice). Our results showed that macrophages from M1^-/-^ mice were more susceptible to HSV-1 infection *in vitro* than were macrophages from WT mice. M1^-/-^ mice were highly susceptible to ocular infection with virulent HSV-1 strain McKrae, while WT mice were refractory to infection. In addition, M1^-/-^ mice had higher virus titers in the eyes than did WT mice. Adoptive transfer of M1 macrophages from WT mice to M1^-/-^ mice reduced death and rescued virus replication in the eyes of infected mice. Infection of M1^-/-^ mice with avirulent HSV-1 strain KOS also increased ocular virus replication and eye disease but did not affect latency-reactivation seen in WT control mice. Severity of virus replication and eye disease correlated with significantly higher inflammatory responses leading to a cytokine storm in the eyes of M1^-/-^ infected mice that was not seen in WT mice. Thus, for the first time, our study illustrates the importance of M1 macrophages specifically in primary HSV-1 infection, eye disease, and survival but not in latency-reactivation.

## Author summary

Macrophages circulating in the blood or present in different tissues constitute an important barrier against infection. We previously showed that the absence of M2 macrophages does not impact HSV-1 infectivity *in vivo*. However, in this study we demonstrated an essential role of M1 macrophages in protection from primary HSV-1 replication, death, and eye disease but not in latency-reactivation.

## Introduction

HSV-1 infection impacts global populations with mild to severe health complications. The virus establishes lifelong latent infections in neurons, but reactivation can be triggered in response to a variety of stimuli [1,2]. The HSV-1 life cycle involves an initial pre-clinical innate immune response phase that controls the severity of infection and is characterized by neutrophils, macrophages, NK cells, dendritic cells, NKT cells, and their functions [3]. The role of the adaptive immune response in protecting against ocular HSV-1 infection and disease had been extensively studied [4], but little is known about the role of the early host innate immune response in controlling the severity of infection and disease. Macrophages are innate cell responders and are known to be early-responders to HSV-1 infection along with other innate immune cells [5,6]. We previously investigated the time at which various immune cells infiltrated the cornea of infected mice and found that macrophages with M1 phenotype were the dominant corneal infiltrates as early as 1 hour post ocular infection [5]. Macrophages are classified as M1 and M2 phenotypes based on the environmental cues [7,8]. Cytokines like IFNγ or GM-CSF promote differentiation of total macrophages toward M1 polarization whereas, cytokines like IL-4 or M-CSF promote macrophage differentiation toward M2 polarization [9,10]. A proper equilibrium between the M1 and M2 macrophage subtypes must be maintained to avoid detrimental host states leading to various diseases and inflammatory conditions [11].

M1 phenotypes are believed to be involved in initiating and advancing inflammatory disease conditions whereas, M2 phenotypes are associated with the resolution and repairing phases of inflammation [12,13]. Our previous work showed that injection of IFNγ DNA into wild-type (WT) mice increased primary and latent infection in latently infected mice, while injection of CSF-1 DNA reduced primary and latent infection in these mice [9]. Similarly, we have shown that ocular infection of WT mice with a recombinant virus expressing IL-4 (HSV-IL-4), which promoted macrophage responses toward M2, was more efficacious against both primary and latent infection than mice infected with a recombinant virus expressing IFNγ (HSV- IFNγ) [10].

We recently showed that although M2 macrophages are involved in resolving inflammatory diseases, absence of the macrophage M2 population (M2^-/-^ mice), did not have a major impact on viral replication, latency-reactivation, or eye disease after ocular HSV-1 infection and infected mice behaved similar to wild-type mice. In contrast, mice overexpressing M2 macrophages (M2-OE mice) had significantly higher primary viral replication, phagocytic activity, and latency [14]. Our results showed that it is important to maintain a homeostatic balance of M1 and M2 macrophage populations. After conceptualizing M2 phenotype functions and possible side-effects of M1 macrophages, we asked what role, if any, the absence of M1 macrophages may play in HSV-1 infection. Thus, we used STAT1 (signal transducers and activators of transcription-1) conditional knockout mice lacking M1 macrophages (M1^-/-^ mice in this study) [15]. In our previous studies we pushed macrophage responses toward M1 in the presence of M2 or vice versa [9,10]. Similar to our M2^-/-^ study [14], we also evaluated the effect of absence of M1 (and presence of M2) phenotypes on primary viral replication, eye disease, and latency-reactivation using M1^-/-^ mice.

In our current study, we found that M1^-/-^ mice were highly susceptible to infection with virulent HSV-1 strain McKrae, while WT control mice were refractory to infection. Virus replication was also significantly higher in the eyes of M1^-/-^ infected mice and in their bone marrow (BM)-derived macrophages than in control WT mice. Infected M1^-/-^ BM-derived macrophages had significantly less expression of 24 of 32 tested cytokines and chemokines than did control WT infected macrophages. We also found that corneas of M1^-/-^ infected mice had significant upregulation of inflammatory pathways compared to control mice. Adoptive transfer of WT mouse bone marrow M1 macrophages to M1^-/-^ mice partially restored survival and ocular virus replication in recipient M1^-/-^ mice to levels similar to those of control WT mice. To confirm our finding that the absence of M1 macrophages augments disease severity, we showed that after infection with avirulent HSV-1 strain KOS, M1^-/-^ mice were protected from death but still had significantly higher virus replication in the eyes and more eye disease than did WT mice. However, levels of latency and reactivation were similar in M1^-/-^ and control mice. Hence, our results demonstrate that M1 macrophages are essential to control primary virus infection, survival, and eye disease but not latency-reactivation.

## Results

### Effect of HSV-1 infection on NOS2 and Arg1 expression in bone marrow-derived macrophages from M1^-/-^ mice

**Fig 1:**
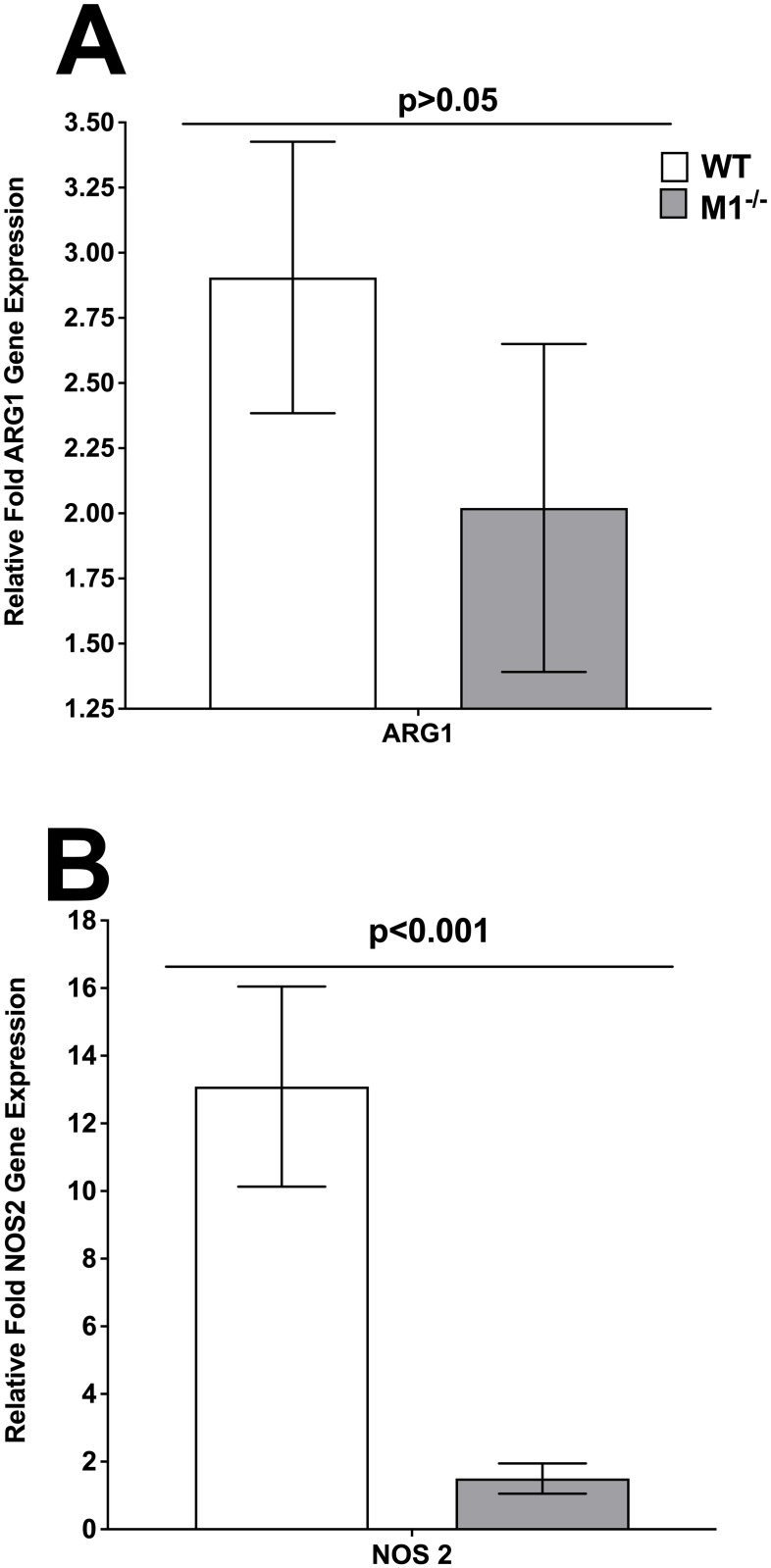
Validation of macrophage phenotype. Bone marrow-derived macrophages from WT and M1^-/-^ mice were polarized into M1 and M2 phenotypes as described in Materials and methods. The cells were then infected with 1 pfu/cell of HSV-1 strain McKrae for 1 hr and harvested 24 hr PI. Total RNA was isolated and subjected to TaqMan qRT-PCR using ARG-1 (M2 marker) and NOS2 (M1 marker) specific primers. Expression of ARG1 and NOS2 mRNA was normalized to that of GAPDH RNA. Each bar represents the mean ± SEM from two independent experiments (N = 4). Panels: A) ARG1; and B) NOS2.

Macrophages display great plasticity and can change their function in response to different stimuli and based on their activation state are divided to M1 and M2 [7]. Currently, there is no *in vivo* markers for M1 and M2 identification, while NOS2 and ARG1 had been used widely as markers of M1 and M2 for *in vitro* studies, respectively [9,16–18]. To determine the effect of HSV-1 infection on NOS2 and ARG1 expression, BM cells were isolated from WT and M1^-/-^ mice and cultured *in vitro* with IL-4 to generate M2, or IFNγ to generate M1, macrophages. M1 and M2 macrophages were harvested on day 6 post treatment and infected with 1 pfu/cell of HSV-1 strain McKrae for 24 hr. Total RNA was isolated and expression of NOS2 and ARG1 were measured by qRT-PCR (Fig 1). ARG1 levels were similar in macrophages isolated from WT and M1^-/-^ mice (Fig 1A, p>0.05) suggesting that the absence of M1 macrophages does not affect ARG1 expression. As expected, NOS2 levels were significantly lower in M1^-/-^ mice than in WT mice (Fig 1B, p<0.001), suggesting that the absence of M1 macrophages in M1^-/-^ mice affects NOS2 but not ARG1 expression in isolated macrophages. These results confirm a previously published study showing that the absence of STAT1 in macrophages reduced NOS2 but not ARG1 expression [15,19].

### Macrophages isolated from M1^-/-^ mice are more susceptible to HSV-1 infection than macrophages from WT mice

Previously we reported that macrophages isolated from signal transducers and activators of transcription-1 deficient (STAT1^-/-^) mice were more susceptible to HSV-1 replication than were macrophages from different strains of WT mice [20]. In this study we tested if macrophages from M1-deficient mice similar to macrophages from STAT1^-/-^ mice are susceptible to HSV-1 infection. Thus, we isolated BM from WT and M1^-/-^ mice. After isolation, macrophages were infected with 0.1 pfu/cell or 1 pfu/cell of HSV-1 strain McKrae for 12, 24, and 48 hr. As a control, Rabbit skin (RS) cells were infected with 0.1 pfu/cell or 1 pfu/cell of HSV-1 strain McKrae for 24 and 48 hr. The kinetics of virus replication were quantitated by determining the amount of infectious virus at various times post-infection using a plaque assay.

**Fig 2:**
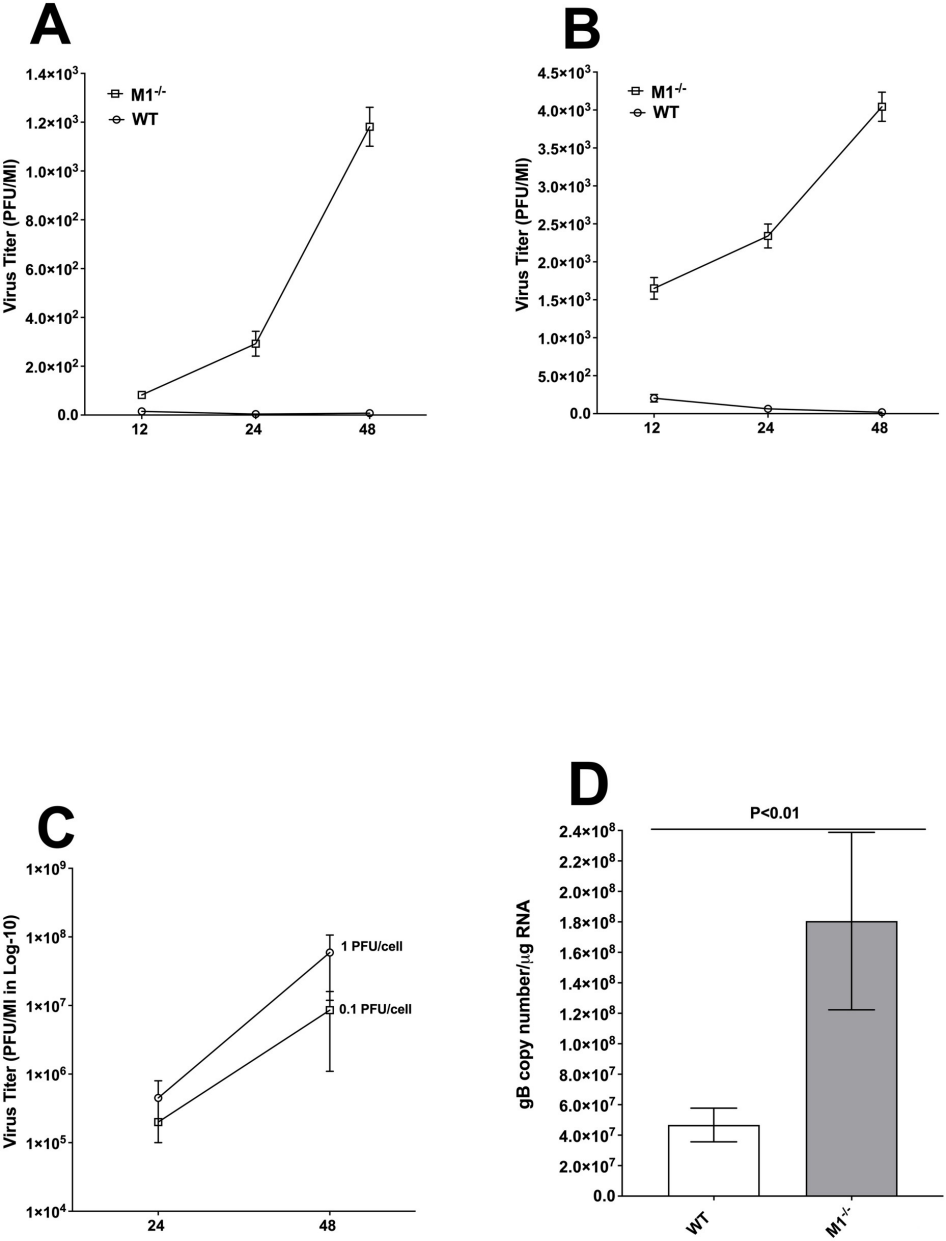
Replication of HSV-1 in macrophages isolated from M^-/-^ mice. (A, B, C) Virus replication. Subconfluent monolayers of macrophages derived from M1^-/-^ and WT mice were infected with 0.1 or 1 pfu/cell of HSV-1 strain McKrae for 12, 24, or 48 hr as described in Materials and methods. Similarly, subconfluent monolayers of RS cells were infected with 0.1 or 1 pfu/cell of McKrae virus for 24 and 48 hr. Virus replication in macrophages infected with 0.1 pfu/cell of McKrae (Panel A); 1 pfu/cell McKrae virus (Panel B); in RS cells infected with 0.1 and 1 pfu/cell of McKrae virus (Panel C); and gB transcript in infected macrophages (Panel D) was determined at indicated times by standard plaque assays. Each point represents the mean ± SEM from two independent experiments (N = 4). D) gB transcript in infected macrophages. Bone marrow-derived macrophages from WT and M1^-/-^ mice were infected with 1 pfu/cell of McKrae for 24 hr. Total RNA was isolated and TaqMan RT-PCR was performed using gB-specific primers as described in Materials and methods. Estimated relative copy number of HSV-1 gB was calculated using standard curves generated from pAC-gB1. Briefly, DNA template, serially diluted 10-fold such that 5 μl contained from 10^3^ to 10^11^ copies of LAT, was then subjected to TaqMan RT-PCR with the same primer set. The copy number for each reaction was determined by comparing the normalized threshold cycle of each sample to the threshold cycle of the standard. GAPDH expression was used as a normalization control.

Virus replication did not differ significantly between cells infected with M1^-/-^ or WT virus at 12 hr post-infection (PI) (Fig 2A, p>0.05), but M1^-/-^ cells displayed significantly more virus replication at 24 and 48 hr PI (Fig 2A, p<0001, at both time points). Virus replication in M1^-/-^ cells infected at 1 pfu/cell, was significantly higher at 12, 24, and 48 hr PI than seen in WT infected cells (Fig 2B, p<0.001). At both time points (24 and 48 hr PI) and dose of infection (0.1 pfu/cell or 1 pfu/cell), virus replicated more efficiently in RS cells and the differences were not statistically significant between time points (Fig 2C, p = 0.5, at both time points). These results showed that the absence of M1 macrophages leads to higher virus replication than in WT infected macrophages. Thus, similar to macrophages from STAT1^-/-^ mice, the absence of M1^-/-^ in macrophages also makes them more susceptible to infection than macrophages from WT mice. Similar to this study, we previously reported that macrophages polarized toward the M1 state had less virus replication than in M2 polarized macrophages [9].

To further confirm these results, infected macrophages from WT and M1^-/-^ mice were infected with 1pfu/cell of HSV-1 and infected cells were harvested 24 hr PI. Total RNA was isolated from infected cells and subjected to TaqMan qRT-PCR to estimate levels of gB mRNA. GAPDH mRNA level was used as an internal control. The results showed significantly higher gB copy number in macrophages from M1^-/-^ mice than in those from WT mice (Fig 2D, p<0.01). These results suggest that the absence of STAT1 in M1 macrophages has a profound effect on virus replication and is indispensable for control of virus replication.

### Detection of GFP expression in infected macrophages by FACS

**Fig 3:**
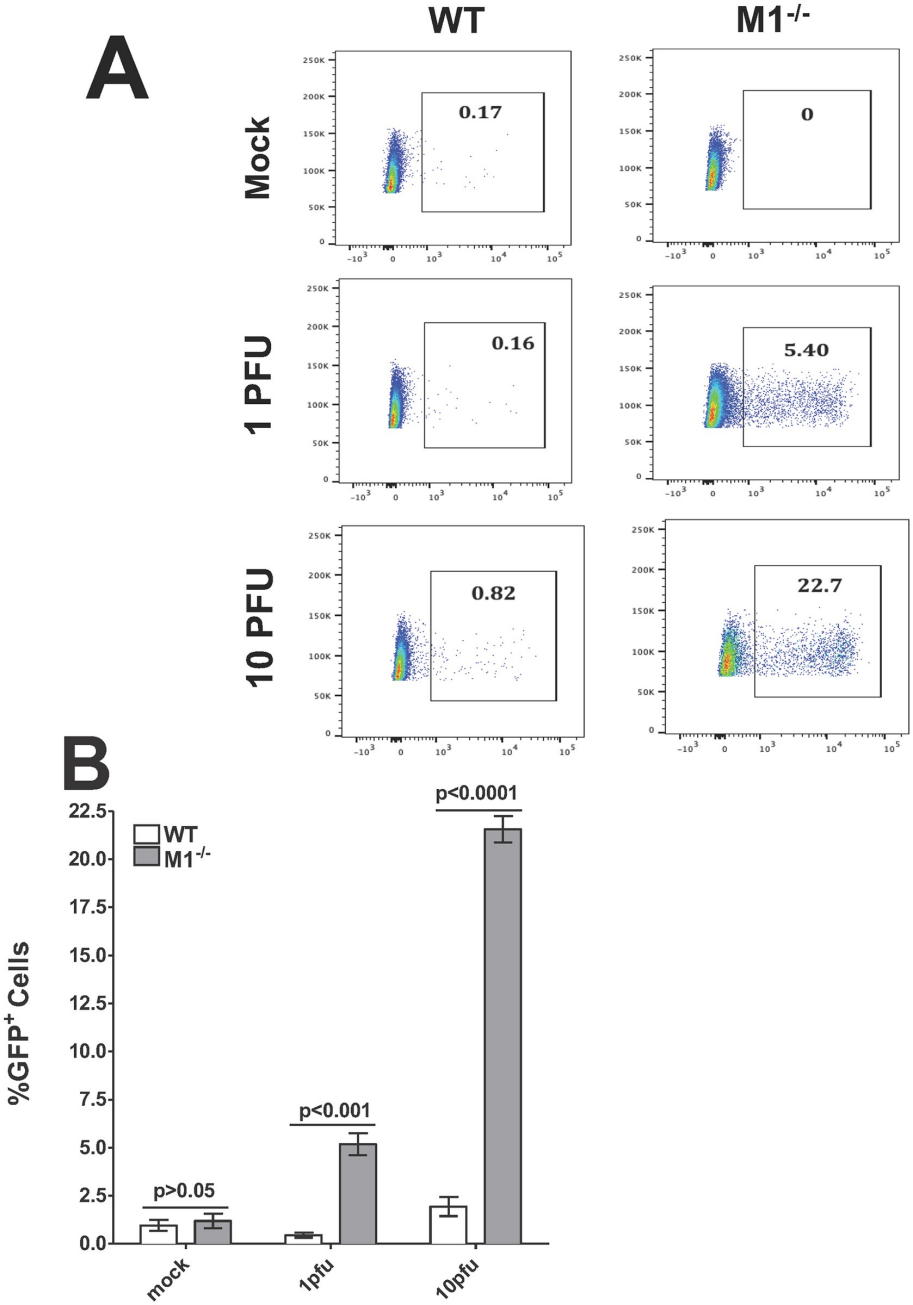
FACS analyses of isolated macrophages. Subconfluent monolayers of macrophages isolated from M1^-/-^ and WT mice were infected with 1 or 10 pfu/cell of HSV-GFP^+^ virus. At 24 hr PI, cells were harvested for FACS analysis. The percent of cells positive for GFP-expression are shown. Panels: **A)** Representative plots of HSV-GFP^+^ infected with 1 or 10 pfu/cell or mock-infected macrophages; and **B)** Percentage plots of HSV-GFP^+^ infected or mock-infected macrophages and quantified by FACS. Each point represents the mean ± SEM from two independent experiments (N = 4).

To confirm that macrophages isolated from M1^-/-^ mice are permissive to HSV-1 replication, monolayers of macrophages isolated from M1^-/-^ and wild-type control mice were infected with 1 pfu or 10 pfu/cell of HSV-GFP^+^ virus or were mock-infected. The kinetics of virus replication were quantified by FACS analysis using the GFP tag to determine the percent GFP^+^ infected cells. We found that M1^-/-^ infected macrophages had a significantly higher percent of GFP-positive cells at 1 pfu/cell (Fig 3A, 5.40% in M1^-/-^ vs 0.16% in WT, 1 pfu) and the level of GFP-positive cells after infection with 10 pfu/cell of virus increased to 22.7% in M1^-/-^ infected macrophages with only 0.82% GFP-positive cells in the WT macrophage control group (Fig 3A, 10 pfu). No significant differences were detected between mock-infected M1^-/-^ and WT macrophages (Fig 3A, Mock). The average percent GFP-positive WT and M1^-/-^ macrophages with and without infection described above was determined from three separate experiments. The percent GFP-positive cells in mock-infected WT and M1^-/-^ macrophages was similar (Fig 3B, p>0.05). M1^-/-^ macrophages infected with either 1 pfu (Fig 3B, p<0.001) or 10 pfu (Fig 3B, p<0.0001) had significantly more GFP-positive macrophages than WT mice infected with similar amounts of virus. Thus, FACS analysis confirmed our results for viral replication and viral mRNA expression, suggesting that STAT1 contributes to the resistance of WT macrophages to HSV-1 replication but not infection.

### M1^-/-^ mice display compromised phagocytic function with enhanced virus dose

**Fig 4:**
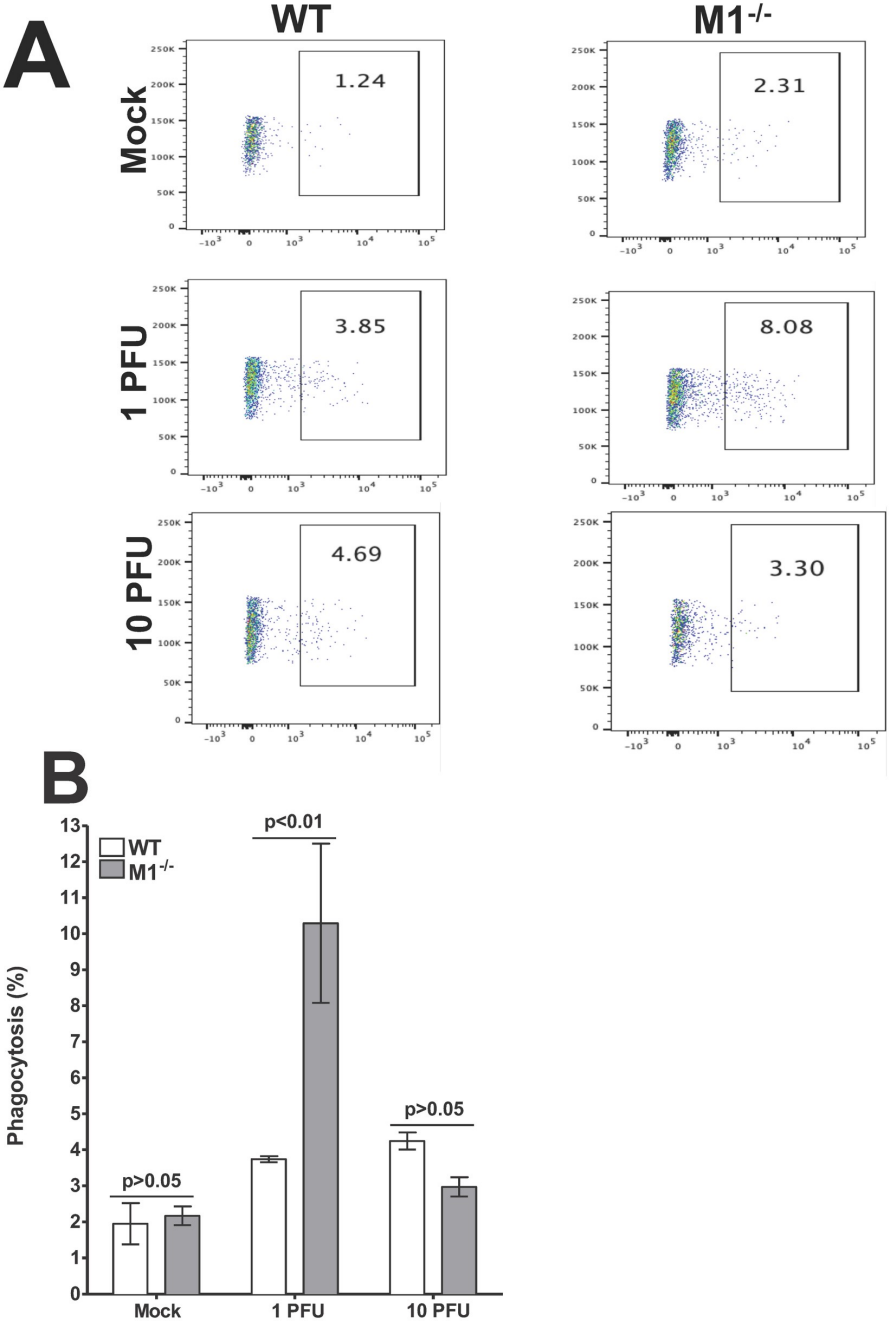
Phagocytosis assay on *in vitro* derived bone marrow-derived (BM) macrophages. Bone marrow cells from WT and M1^-/-^ mice were cultured and differentiated into macrophages with appropriate culture conditions as described in Materials and methods. After generating macrophages, adhered cells were infected with 1 or 10 pfu/cell of McKrae virus for 1 hr. Infected cells were harvested 24 hr PI and incubated with latex beads complexed with rabbit IgG-FITC. Cells were stained with F4/80 AF 564 antibody and analyzed by flow cytometry. Panels: **A)** Representative plots of WT and M1^-/-^ BM cells; and **B)** Percentage phagocytosis plots from three separate experiments.

Along with neutrophils and dendritic cells, macrophages are also professional phagocytes as they internalize and kill invading pathogens, a process known as phagocytosis [21]. Bone marrow-derived macrophages were isolated from WT and M1^-/-^ mice and infected with HSV-1 strain McKrae or mock-infected and incubated with FITC labeled beads for 2 hr as described in Materials and methods. The cells were then scraped out and collected in FACS tubes and stained with F4/80 antibody. The extent of phagocytosis was measured by flow cytometry using a phagocytosis assay kit (IgG-FITC). The number of FITC positive cells was significantly higher in M1^-/-^ macrophages infected with 1 pfu/cell of HSV-1 than in macrophages isolated from WT mice (Fig 4A, 1 pfu, 8.08% in M1^-/-^ group vs 3.85% in WT group). In contrast, the number of FITC positive cells after HSV-1 infection with 10 pfu/cell in M1^-/-^ infected macrophages was lower than in WT infected macrophages (Fig 4A, 10 pfu, 3.3% in M1^-/-^ group vs 4.69% in WT group). The mock group showed no significant differences between the WT and M1^-/-^ mice (Fig 4A, Mock, 1.24% for WT macrophages vs 2.31% for M1^-/-^ macrophages).

The average percentage of FITC positive cells from WT and M1^-/-^ cells infected with 1 or 10 pfu/cell of HSV-1 or mock-infected was determined from three separate experiments. Percent phagocytosis in mock-infected WT and M1^-/-^ cells were similar (Fig 4B, p>0.05, Mock). Percent phagocytosis in M1^-/-^ cells after infection with 1 pfu/cell of HSV-1 was significantly higher than in WT infected cells (Fig 4B, M1^-/-^ versus WT group, p<0.01, 1 pfu). In contrast, after infection of M1^-/-^ cells with 10 pfu/cell of HSV-1 the level of phagocytosis declined to levels similar to those of WT infected cells (Fig 4B, M1^-/-^ versus WT group, p>0.05, 10 pfu). These results demonstrate that phagocytosis is enhanced in macrophages isolated from M1^-/-^ mice after infection with 1 pfu/cell but declines when cells are infected with 10 pfu/cell of HSV-1. This reduced phagocytosis could be due to high dose of virus infection (10 pfu/cell) which may have shot down the host immune response leading to lower phagocytosis. Complementing this study, we previously showed that higher levels of M2 macrophages are associated with higher phagocytosis [14].

### Macrophages from M1^-/-^ mice after infection have higher chemokine and cytokine expression compared to macrophages from WT mice

Results described above in Figs 3 and 4 shown that HSV-1 replicates more efficiently in macrophages isolated from M1^-/-^ mice than in macrophages from WT mice. To determine if the absence of M1 macrophages affects cytokine and chemokine production after HSV-1 infection, macrophages isolated from WT and M1^-/-^ mice were infected with HSV-1 strain McKrae as described in the Materials and methods. Twenty-four hours PI, cytokine and chemokine levels were determined in culture media collected from wells containing infected cells using a Luminex bead-based multiplex cytokine profiling assay as we described previously [9,22] (Table 1). M1^-/-^ infected macrophages had significantly higher levels of GM-CSF, IL-1α, IL-1β, IL-5, IL-6, IL-9, IL-10, IL-12 (p40), IL-12 (p70), LIF, LIX, IP-10, KC, MCP-1, MIP-1α, MIP-1β, MIP-2, MIG, RANTES, TNF-α, and KC than WT infected macrophages (Table 1). GM-CSF and M-CSF levels were higher in M1^-/-^ infected macrophages than WT infected macrophages but the differences were not statistically significant (Table 1, p>0.05). However, levels of Eotaxin, IFNγ, IL-2, IL-4, IL-3, IL-7, IL-15, and VEGF did not differ between WT and M1^-/-^ infected macrophages (not shown). These results suggest that the absence of M1 macrophages alters the expression of many, but not all cytokines and chemokines secreted by M1^-/-^ macrophages. Many of the elevated cytokines and chemokines are pro-inflammatory and may contribute to higher virus replication in M1^-/-^ macrophages than in WT macrophages.

**Table 1:** Cytokine/chemokine detected in HSV-1 infected BM-derived macrophages ^a^.

	Mouse strain		
Cytokine/Chemokine	M1^-/-^	WT	P value
G-CSF	57.2 ± 8.3	35.4 ± 3.2	0.0704
GM-CSF	54.8 ± 3.2	23.7 ± 3.0	0.0021
IL-1α	50.4 ± 4.1	18.8 ± 1.2	0.0018
IL-1β	89.6 ± 6.1	28.4 ± 0.9	0.0006
IL-5	19.7 ± 0.4	3.3 ± 0.3	0.0001
IL-6	3029.0 ± 110.3	379.3 ± 40.8	0.0001
IL-9	109.3 ± 8.8	67.0 ± 5.6	0.0154
IL-10	37.5 ± 6.1	8.0 ± 0.7	0.0086
IL-12 (p40)	10.0 ± 1.4	1.9 ± 0.3	0.0048
IL-12 (p70)	5.0 ± 0.5	3.2 ± 0.0	0.0228
LIF	1.6 ± 0.4	0.2 ± 0.2	0.0352
IL-13	42.7 ± 4.3	9.6 ± 0.6	0.0016
LIX	61.9 ± 4.7	32.0 ± 7.6	0.0287
IP-10 (CXCL10)	10871.7 ± 358.3	3285.7 ± 131.2	0.0001
KC (CXCL1)	679.3 ± 28.5	384.9 ± 9.8	0.0006
MCP-1 (CCL2)	9639.3 ± 422.5	1084.8 ± 74.3	0.0001
MIP-1α (CCL3)	1542.0 ± 69.5	640.2 ± 13.8	0.0002
MIP-1β (CCL4)	24402.7 ± 6150.8	2461.7 ± 47.4	0.0234
M-CSF	9.5 ± 1.3	6.1 ± 0.3	0.0634
MIP-2 (CXCL2)	2649.3 ± 103.9	1332.0 ± 72.3	0.0005
MIG (CXCL9)	1199.3 ± 55.7	7.4 ± 0.8	0.0001
RANTES (CCL5)	610.7 ± 91.7	68.1 ± 2.6	0.0041
TNF-α	126.8 ± 10.2	67.1 ± 4.3	0.0057

^**a**^Cytokine/chemokine levels in culture media were analyzed using mouse 32-plex panels and are shown as pg/ml. Experimental procedures are described in Materials and methods. Briefly, isolated macrophages from BM were infected with 10 pfu/cell of HSV-1 strain McKrae for 1 h at 37°C, washed with PBS, and incubated for an additional 24 h in fresh media. Infected cell supernatants were collected. Levels of Eotaxin, IFNγ, IL-2, IL-4, IL-3, IL-7, IL-15, and VEGF did not differ between WT and M1^-/-^ infected macrophages. Results indicate mean ± SEM (n = 3). In contrast to infected macrophages described above, cytokines and chemokines levels in mock infected macrophages for both M1^-/-^ and WT groups was below detection level.

### Absence of M1 macrophages leads to higher virus replication in mouse eyes

**Fig 5:**
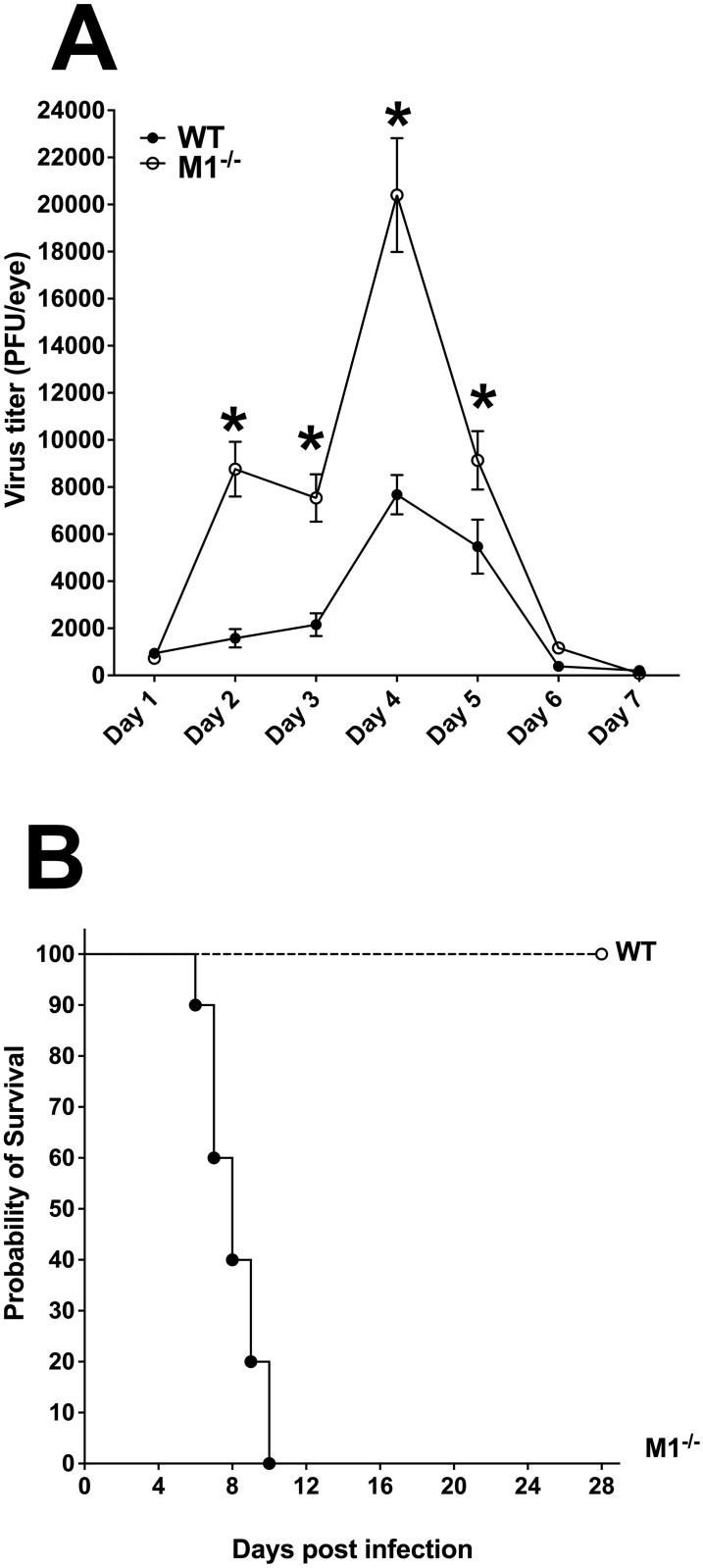
Virus replication and survival of M1^-/-^-infected mice. A) Virus replication in tears of infected mice. WT and M1^-/-^ mice were ocularly infected with 2 X 10^5^ pfu/eye of McKrae virus without corneal scarification. Tear films were collected on days 1–7 PI and virus titers were determined by standard plaque assays as described in Materials and methods. Each point represents the mean ± SEM of 20 eyes per group. p-values less than 0.05 were considered statistically significant and marked by a single asterisk (*); and B) Absence of M1 macrophages enhanced mortality in infected mice. Survival in the above ocularly infected mice was monitored for 28 days. The graph represents survival from two different experiments.

To determine the effects of absence of M1 macrophages on HSV-1 replication *in vivo*, WT and M1^-/-^ mice were ocularly infected with 2 X 10^5^ pfu/eye of virulent HSV-1 strain McKrae. Tear films were collected from day 1 to day 7 PI from ten mice per group and viral titers were determined by standard plaque assay as described in Materials and methods. Infected M1^-/-^ mice had significantly higher viral titers in their eyes from days 2–5 PI than did WT mice (Fig 5A, p<0.05 for all 4 points). A peak of virus replication was observed on day 4 PI in both WT and M1^-/-^ mice and declined thereafter. The effect of M1 macrophage absence on survival of infected mice was evaluated. All M1^-/-^ infected mice died by day 10 PI, whereas all of infected WT mice survived ocular infection (Fig 5B, p<0.001, Fisher exact test). These results suggest that M1^-/-^ mice are highly susceptible to HSV-1 infection and that M1 plays a crucial role in controlling virus replication in the eye. Similar results were reported for STAT1-knockout mice, with STAT1 completely deleted, while in this study M1^-/-^ mice lack STAT1 only in macrophages [23].

### Adoptive transfer of M1 macrophages rescues virus replication in the eye and improves survival and eye disease in infected M1^-/-^ mice

**Fig 6:**
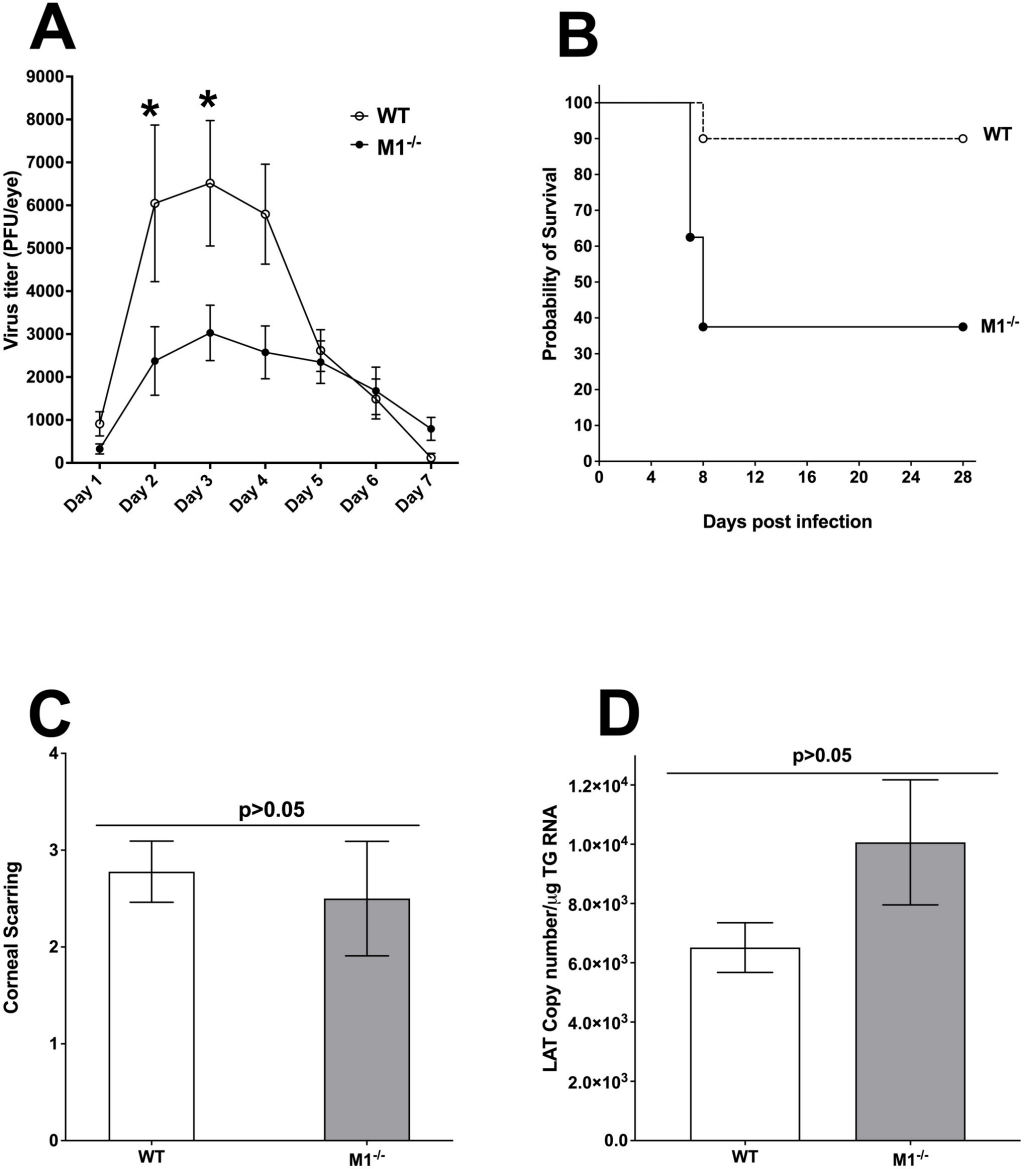
Virus replication, survival, corneal scarring (CS) and LAT expression after adoptive transfer of M1 macrophages to M1^-/-^ recipient mice. A) Virus replication in tears of infected mice. WT and M1^-/-^ mice, after receiving M1 macrophages from WT mice, were ocularly infected with 2 X 10^5^ pfu/eye of virulent HSV-1 strain McKrae. Tear films were collected from ocularly infected mice on days 1–7 PI and virus titers were determined by standard plaque assays as described in Materials and methods. Each point represents the mean + SEM of 20 eyes per group: B) Survival of M1^-/-^ recipient mice. Survival in the above infected mice was monitored for 28 days and the graph represents the average results from two different experiments using 9 WT mice and 3 M1^-/-^ recipient mice; C) CS in M1^-/-^ recipient mice. Corneal scarring (CS) in surviving mice were examined on day 28 PI as described in Materials and methods. CS scores represent the average ± SEM from 6 eyes for M1^-/-^ mice and 18 eyes for WT mice; and D) Latency levels in the TG of latently infected M1^-/-^ recipient mice were analyzed. On day 28 PI, individual TG from each mouse were harvested and quantitative RT-PCR was performed. In each experiment, estimated relative LAT copy number was calculated using standard curves generated from pGEM-5317. Briefly, DNA template was serially diluted 10-fold such that 5 μl contained from 10^3^ to 10^11^ copies of LAT, then subjected to TaqMan PCR with the same set of primers. The copy number for each reaction was determined by comparing the normalized threshold cycle of each sample to the threshold cycle of the standard. GAPDH expression was used to normalize relative LAT RNA copy number in the TG. LAT copy number represents the average ± SEM from 6 TG for M1^-/-^ mice and from 18 TG for WT mice; p-values were determined using one-way ANOVA.

Because the absence of M1 macrophages in M1^-/-^ mice makes them highly susceptible to ocular infection with virulent HSV-1 strain McKrae (Fig 5), we tested if adoptive transfer of M1 macrophages from WT mice to M1^-/-^ mice could rescue the phenotype of M1^-/-^ mice. Bone marrow from WT mice were isolated and polarized toward M1 macrophages using IFNγ treatment as we described previously [9,10]. M1 generated macrophages were adoptively transferred to M1^-/-^ mice by tail vein injections and 2 hr hours later, recipient M1^-/-^ mice were ocularly infected with 2 X 10^5^ pfu/eye of virulent HSV-1 strain McKrae. WT mice were similarly infected and used as controls. Virus replication, percent survival, corneal scarring, and latency were recorded. Tear films from ten infected mice per group were collected from days 1 to 7 PI and virus titers were determined by standard plaque assays as described in Materials and methods. Virus titers in the eyes of recipient M1^-/-^ mice on days 2 and 3 were lower than in WT mice (Fig 6A, day 2–3 PI, p<0.05). Also, virus titers in M1^-/-^ mice on day 4 was lower than in WT mice but the differences were not statistically significant (Fig 6A, day 4 PI, p>0.05). Thus, compared with results shown in Fig 5A, adoptive transfer of M1 macrophages from WT mice to recipient M1^-/-^ mice significantly reduced virus replication in the eyes of infected mice compared with non-recipient M1^-/-^ mice and WT mice. In addition, lower virus replication in M1 recipient M1^-/-^ mice than in WT mice on days 2–4 post ocular infection could be due to a protective role of transferred macrophages at early times PI.

Survival of WT mice and M1^-/-^ mice that received WT-derived M1 macrophages (Fig 6A) was monitored for 28 days with 35% of M1^-/-^ mice that received an M1-macrophage transfer and 90% of control mice, surviving ocular infection (Fig 6B, p<0.05, Fisher exact test). Survival between M1^-/-^ mice that did or did not receive a macrophage transfer was statistically significant (compare M1^-/-^ mice in Fig 5B with M1^-/-^ mice in Fig 6B, p<0.05, Fisher exact test). This suggests that reduced mortality in M1^-/-^ recipient mice is due to the transfer of M1 macrophages from WT mice rather than from another defect associated with conditional STAT1 knockout in macrophages. Mice generally die between days 6–9 PI from encephalitis, at which time most of the transferred macrophages could have died. The half-life of macrophages *in vivo* is short, which is likely why the transfer did not completely rescue survival but did rescue virus replication in the eye.

To determine the effect of macrophage transfer to M1^-/-^ mice on eye disease, the severity of corneal scarring (CS) was evaluated on days 7, 14, and 28 PI in surviving mice following ocular infection of mice described in Fig 6. CS was also examined in WT control mice infected similarly. CS in recipient M1^-/-^ mice was similar to that in WT mice at all three time points and data for day 28 PI is shown in Fig 6C (P>0.05). Thus, partial rescue of survival in recipient M1^-/-^ mice did not affect CS when compared to WT mice.

To determine the effect of M1 macrophage transfer on HSV-1 latency, TG from surviving latently infected WT and M1^-/-^ recipient mice were isolated on day 28 PI. Latency in individual mouse TG was determined by qRT-PCR using primers from the LAT region of the HSV-1 genome. LAT RNA copy number in TG of WT and M1^-/-^ recipient mice determined as described in Materials and methods, were similar in these mice (Fig 6D; p>0.05). Thus, the level of latency in TG of infected M1^-/-^ recipient mice was similar to that of WT mice. Taken together, data presented in Fig 6 suggest that transfer of M1 macrophages from WT mice to M1^-/-^ mice partially rescued survival, and completely rescued virus replication in the eye, CS, and latency compared with WT control mice.

### Effect of avirulent HSV-1 strain KOS virus on virus replication, survival, eye disease, latency, and reactivation in M1^-/-^ mice

**Fig 7:**
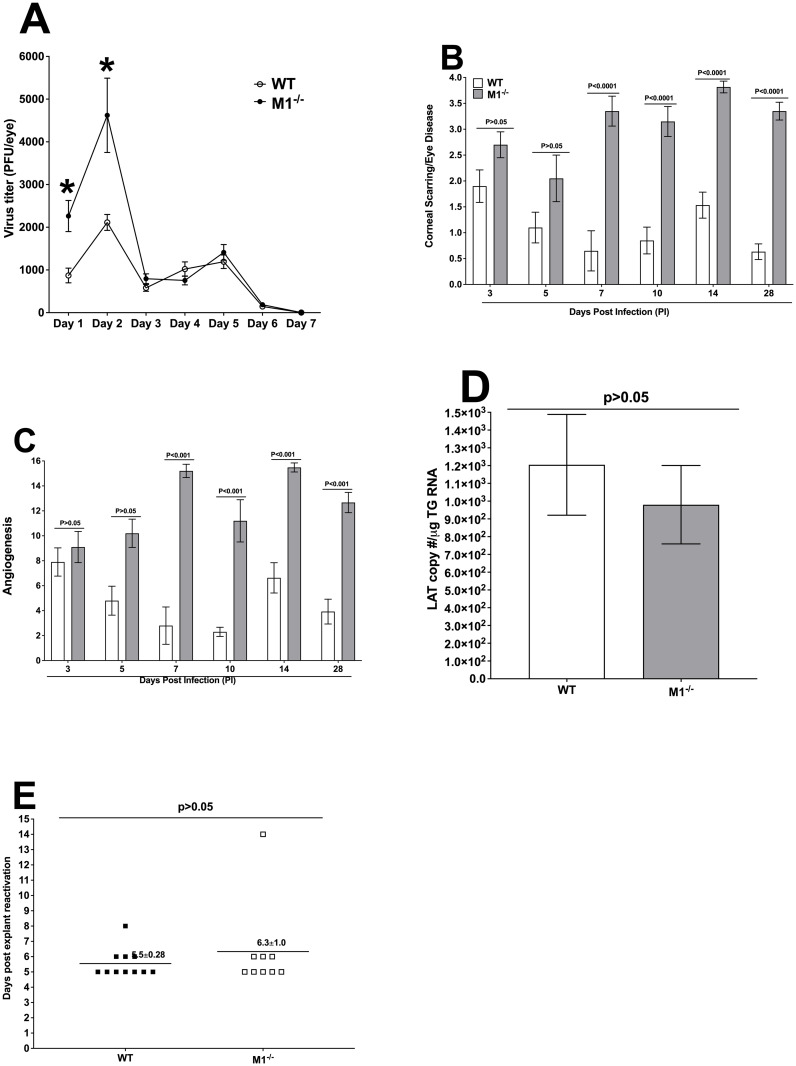
Virus replication, eye disease, latency, and reactivation after infection with avirulent HSV-strain KOS virus. WT and M1^-/-^ mice were ocularly infected with 2 X 10^5^ pfu/eye of avirulent HSV-1 strain KOS following corneal scarification as described in Materials and methods. A) Virus replication in tears of infected mice. Tear films were collected on days 1–7 PI and virus titers were determined by standard plaque assays as described in Materials and methods. Each point represents the mean ± SEM of 32 eyes per group: B and C) Eye disease. Corneal scarring/corneal disease and angiogenesis were examined on days 3, 5, 7, 10,14 and 28 PI as described in Materials and methods. Scores represent the average ± SEM from 10 eyes on days 3, 5, 7 and 10, while 30 eyes for each group of mice on days 14 and 28 PI. p-values were determined using two-way ANOVA; D) LAT expression in TG of infected mice. To analyze latency levels in the TG of latently infected mice on day 28 PI, 20 TG per mouse group were harvested, and quantitative RT-PCR was performed on individual TG from each mouse. In each experiment, the estimated relative LAT copy number was calculated using standard curves generated from pGEM-5317 as described in Fig 6. GAPDH expression was used to normalize relative LAT RNA expression in the TG. p-values were determined using one-way ANOVA; and E) Explant reactivation in TG from latently infected mice. TG from latently infected mice were individually isolated on day 28 PI. Each individual TG was incubated in 1.5 ml of tissue culture media at 37° C. Media aliquots were removed from each culture daily for up to 5 days and plated on indicator RS cells to assess the appearance of reactivated virus. Results are plotted as the number of TG that reactivated daily. Numbers indicate the average time that TG from each group first showed CPE ± SEM. Reactivation is based on 10 TG for WT mice and 8 TG for M1^-/-^ mice. p-values were determined using one-way ANOVA.

As shown above, M1^-/-^ mice were highly susceptible to virulent HSV-1 strain McKrae and the transfer of M1 macrophages into M1^-/-^ mice produced a phenotype that mostly mimicked the WT phenotype. Therefore, avirulent HSV-1 strain KOS was used to determine how the absence of M1 macrophages affected eye disease, latency, and reactivation. Sixteen WT and M1^-/-^ mice from two separate experiments were ocularly infected with 2 X 10^5^ pfu/eye of KOS virus after corneal scarification. Tear films were collected on days 1 to 7 PI from 16 mice per group and virus titers were determined by standard plaque assays. M1^-/-^ mice had significantly higher viral titers on days 1 and 2 PI than did WT mice (Fig 7A, p<0.05), while virus titers on days 3 to 7 were similar in both mouse strains (P>0.05). These results suggest that similar to infection with McKrae virus, infection of M1^-/-^ mice with the avirulent KOS strain enhances viral replication during early time point PI.

Survival over 4 wk was monitored in two separate experiments using groups of 16 WT or M1^-/-^ mice that had been infected ocularly in both eyes with avirulent strain KOS described above. All 16 infected mice in the WT and M1^-/-^ groups survived ocular infection (p = 1; ANOVA) (not shown). In contrast to infection with virulent HSV-1 strain McKrae, and as expected, the absence of M1 did not alter survival in ocularly infected and WT mice.

To determine the effect of M1 absence on corneal scarring and angiogenesis, KOS virus infected WT and M1^-/-^ mice were scored for corneal scarring/corneal disease and angiogenesis on days 3, 5, 7, 10, 14 and 28 PI as described in Materials and methods. Our data indicates no significant differences on days 3 and 5 PI between the groups (Fig 7B, p>0.05). However, on days 7,10,14 and 28 PI, corneal scarring/corneal disease was significantly higher in M1^-/-^ infected mice than in WT infected mice (Fig 7B, p<0.001). Similar to corneal scarring/corneal disease, angiogenesis was not significant on days 3 and 5 PI between the groups (Fig 7C, p>0.05), while it was significantly higher on days 7, 10, 14 and 28 PI in M1^-/-^ infected mice than in WT infected mice (Fig 7C, p<0.001). These results suggest that absence of M1 macrophages lead to higher levels of pro-inflammatory cytokines and severe eye disease, which is consistent with our previous study showing early detection of pro-inflammatory M1 macrophages in the eye of infected mice [5].

We investigated the impact of the absence of M1 on latency and reactivation in surviving WT and M1^-/-^ mice on day 28 PI. HSV-1 replication in eyes during the first two days of infection was higher in M1^-/-^ than in WT mice (Fig 7A) and M1^-/-^ mice also had more severe eye disease (Fig 7B and 7C). Higher virus replication on days 1 and 2 PI in M1^-/-^ mice may increase latency-reactivation in infected mice. TG from surviving WT and M1^-/-^ mice were harvested on day 28 PI and latency levels were determined by qRT-PCR based on HSV-1 LAT expression as described in the Materials and methods. Unlike elevated viral replication, latency (i.e., LAT expression) was lower in M1^-/-^ mice than in WT mice (Fig 7D, p>0.05). These results suggest that the absence of M1 did not affect the establishment and/or maintenance of latency.

Finally, we asked whether higher virus replication in the eye correlates with faster reactivation in M1^-/-^ mice, in contrast to its lack of correlation with latency levels. Virus reactivation was analyzed by explanting individual TG from infected mice on day 28 PI. Consistent with latency levels, time of reactivation was also similar between WT and M1^-/-^ mice (Fig 7E. p>0.05). Thus, in M1^-/-^ mice the time to explant reactivation correlated with the level of latency.

Overall, the above results following KOS virus infection suggest that the absence of M1 macrophages affects early stages of virus replication and eye disease, but not latency-reactivation. This is in line with the function of the innate immune response to infection in the absence of immunity to the infection [24]. It also confirms the important role of macrophage subtypes in protecting from eye disease [25].

### Possible factors causing mortality in M1^-/-^ mice

M1^-/-^ mice used in this study are in a C57BL/6 background but, in contrast to parental mice, we have shown that M1^-/-^ mice are highly susceptible to infection with virulent HSV-1 strain McKrae but not to the avirulent strain KOS. To determine what factors, contribute to high mortality, we looked at 754 myeloid innate immunity genes to identify cells being recruited into the eye and brain. WT and M1^-/-^ mice were ocularly infected with 2 x 10^5^ pfu/eye of HSV-1 strain McKrae without corneal scarification. Corneas and brain were isolated on day 4 PI (the peak of virus replication in the eye) and total RNA was isolated from three mice per group and analyzed on an nCounter FLEX platform as described in Materials and methods.

**Fig 8:**
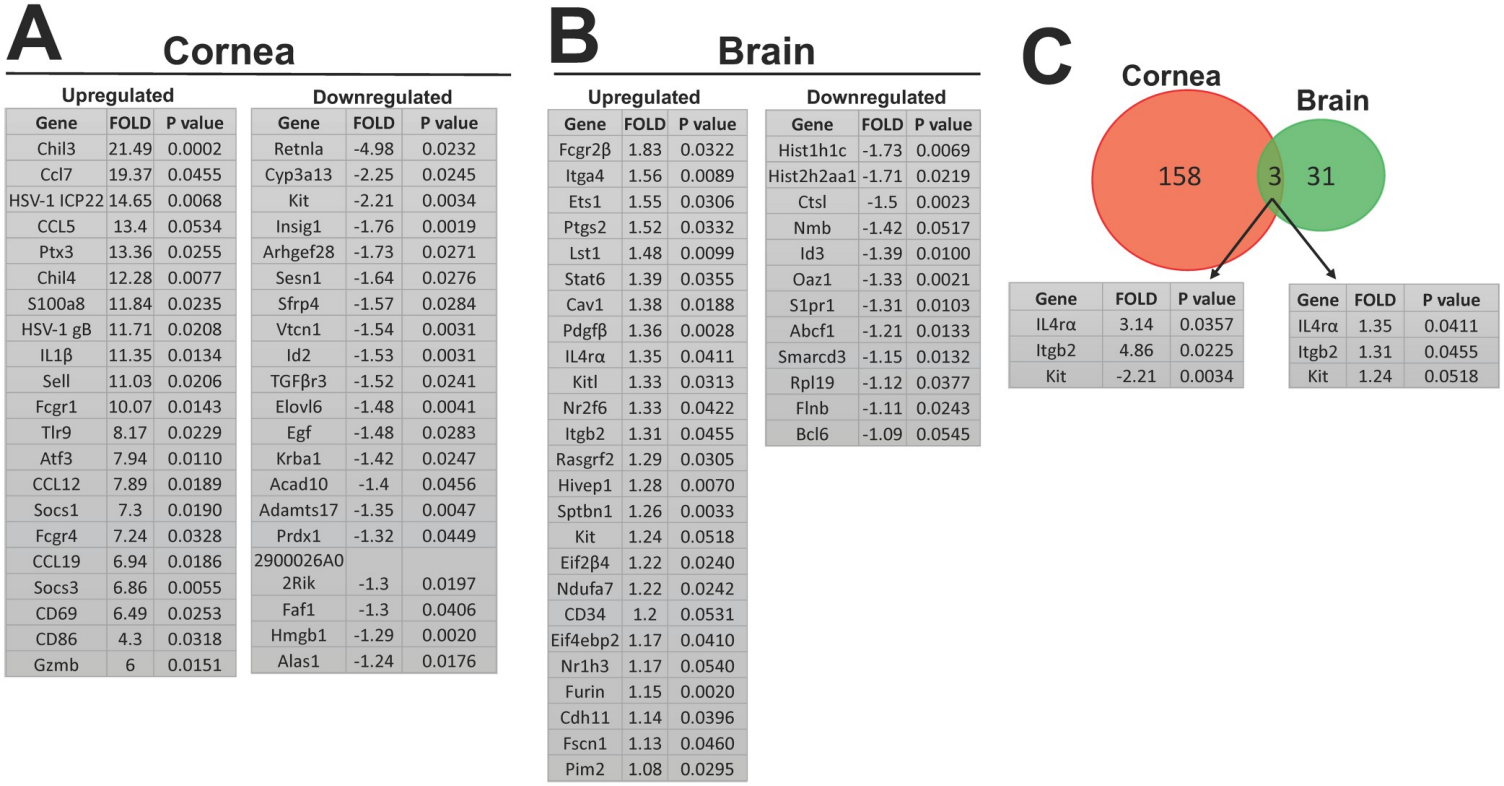
Nanostring gene expression analysis of infected cornea and brain on day 4 PI. WT and M1^-/-^ mice were ocularly infected with 2 X 10^5^ pfu/eye of McKrae virus without corneal scarification. Corneas and brain were collected on day 4 PI and total RNA from pooled corneas per mouse and individual brain were isolated. Total RNA concentration in each well remained at 20 ng/μl. Expression of the 764 gene myeloid immune panel was analyzed as described in Materials and methods. Differentially expressed genes were identified for each group by normalizing samples to housekeeping genes and infected WT mice. A) Upregulated and downregulated genes in cornea after infection. Out of 158 genes totally expressed, the first 22 upregulated genes and the first 20 downregulated genes in infected corneas of M1^-/-^ mice are shown: B) Upregulated and downregulated genes in brain after infection. Out of 31 genes totally expressed, 19 were upregulated, and 12 were downregulated in brain of M1^-/-^ mice when compared to WT infected mice; and C) Venn Diagram showing common genes expressed in corneas and brain of infected mice. IL4rα, Itgb2 and Kit were commonly expressed in corneas and brain of infected mice with different p-values. N = 3 mice corneas or brain.

Differentially expressed genes were identified in WT and M1^-/-^ infected mice by normalizing samples to housekeeping genes in infected WT mice. The list of statistically significant upregulated and downregulated genes in infected corneas (Fig 8A) and brain (Fig 8B) is shown. Of the 158 genes in M1^-/-^ mice, 142 were upregulated in cornea after infection and the first 22 significantly upregulated genes are shown (Fig 8A, all significantly differ from their corresponding genes in WT infected mice, upregulated). The 20 significantly downregulated genes compared to WT mice, are shown in Fig 8A (all significantly differ from their corresponding genes in WT infected mice, downregulated). Of the many upregulated genes in corneas, CCL5 and CD86 were upregulated in M1^-/-^ mice and are known to be M1 markers and are widely expressed in a variety of other cells, e.g., NK cells, dendritic cells, and memory T cells [26,27]. CCL5 also has a role in inflammation, chemotaxis, and immune cell migration [28]. CCL5 are known to act as chemoattractant for T_H_1 cells and other primary immune cells [29]. We also saw significantly higher Chil3 gene expression in corneas from infected M1^-/-^ mice compared to WT mice (21.49-fold) (Fig 8A, upregulated) which is a marker for M2 macrophages along with other M2 markers, e.g. ARG1, Mrc1 and Fizz1 [30]. CCL7 is expressed in various cell types, e.g., stromal cells, airway smooth muscle cells, and keratinocytes, under physiological conditions, and in tumor cells under pathological conditions. CCL7 was upregulated in M1^-/-^ mouse corneas by 9.37-fold relative to WT mouse (Fig 8A, upregulated) after infection and has potent chemoattractant activity for a variety of leukocytes, e,g. monocytes, eosinophils, basophils, dendritic cells (DCs), NK cells, and activated T lymphocytes. CCL7 also recruits a leukocyte subtype to infected tissues to address pathologic invasion and fine-tune the immune response [31]. Most importantly, HSV-1 gB was significantly upregulated in M1^-/-^ mouse corneas (11.71-fold relative to WT) after infection (Fig 8A, upregulated).

ICP22 level was significantly upregulated in M1^-/-^ mouse corneas (14.65-fold relative to WT) after infection (Fig 8A, upregulated). Higher expression of gB and ICP22 transcripts in M1^-/-^ infected mouse corneas also confirm higher virus titers in the eyes of M1^-/^-infected mice. In contrast, expression of HSV-1 gK in infected M1^-/-^ and WT mouse corneas was similar (S1 Fig). To address inflammation activity in infected corneas, IL-1β, a potent pro-inflammatory cytokine [32], was also significantly upregulated in M1^-/-^ mice (11.35-fold) (Fig 8A, upregulated). Gzmb, a pro-inflammatory gene expressed by CD4^+^ cells, mast cells, activated macrophages, neutrophils, basophils, dendritic cells (DCs), and T regulatory cells [33–37], was upregulated by 6-fold in M1^-/-^ infected corneas compared with WT corneas (Fig 8A, upregulated). We found no difference in the expression of Arg1, NOS2, IL-4, IL-6, IFNγ, CD4, CD8, FOXP3, CD25, or Prf1 which are mainly involved in macrophage or T cell identification and differentiation (S1 Fig).

In M1^-/-^ mouse brains, 25 genes were upregulated, and 12 were downregulated relative to WT infected mice (Fig 8B). However, looking at gene expression in the brain, we found minimal differences in upregulated and downregulated gene expression in M1^-/-^ mice relative to that of WT mice (Fig 8B). We saw no significant difference in HSV-1 gB expression in brain (S1 Fig) signifying that virus is not replicating as efficiently in brain as in infected corneas on day 4 PI. Venn diagrams illustrated 158 and 31 upregulated or downregulated genes in cornea and brain of M1^-/-^ mice relative to WT mice (Fig 8C). However, only three common genes [IL-4rα, Itgb2, and Kit were detected in cornea and brain of infected mice (Fig 8C)]. These three common genes followed a contrasting pattern of expression in infected corneas and brain with higher fold difference in corneas and very minor expression in brain relative to WT mice. IL-4 which acts through IL-4rα is an anti-inflammatory cytokine with a crucial role in regulating macrophages [24]. Kit is a tyrosine kinase receptor expressed on some fully differentiated immune cells, e.g. dendritic cells, natural killer cells, and mast cells, highlighting its possible role in the pathogenesis of a wide variety of inflammatory diseases [38]. Finally, ITGβ2 (Integrin Subunit β2) combines with different alpha chains to form different integrin heterodimers and among its related pathways are MET, which promotes cell motility, integrin pathway, blood-brain barrier, and immune cell transmission [39].

**Fig 9:**
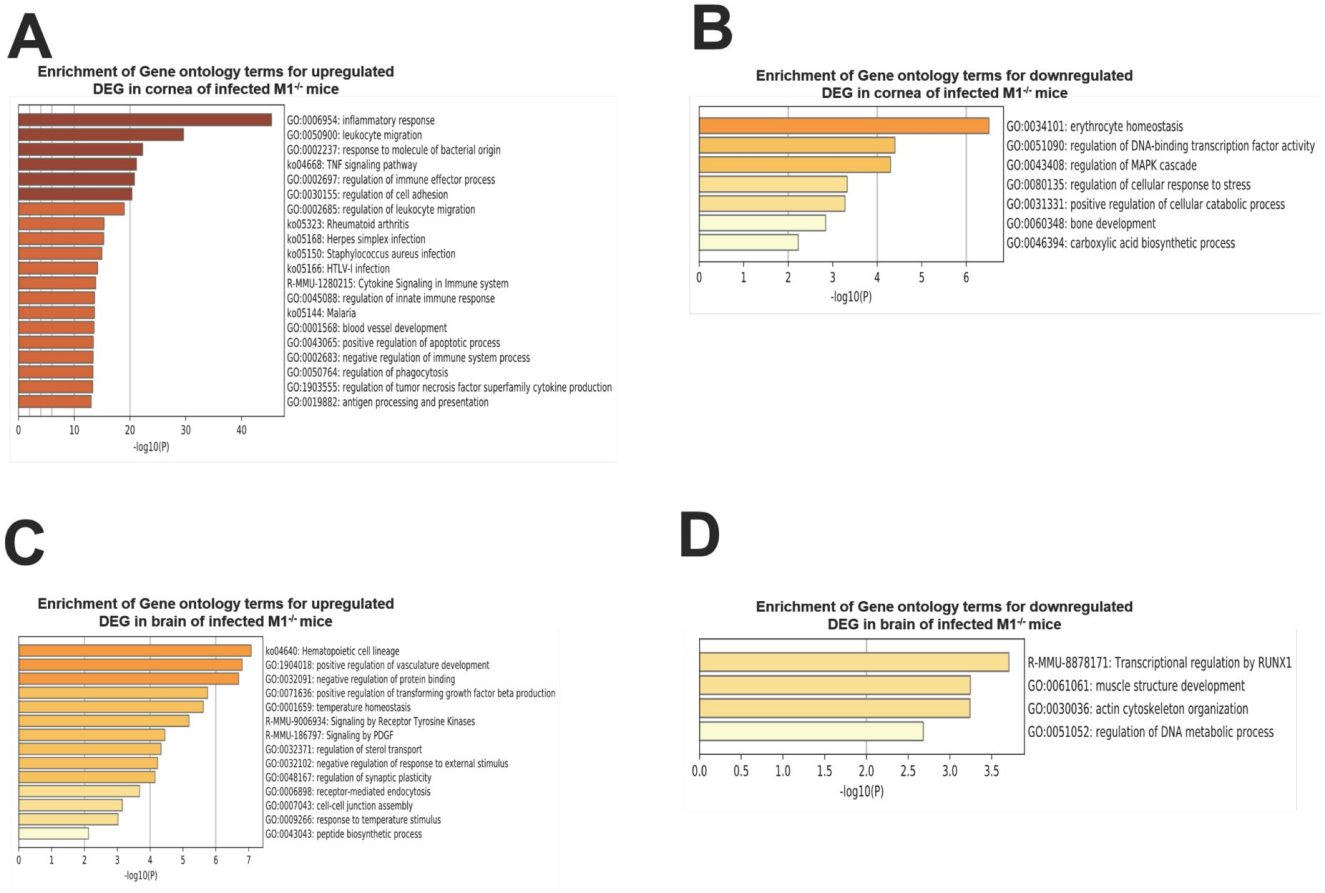
Gene ontology expression in corneas and brain of infected WT and M1^-/-^ mice. A and B) Upregulated and downregulated pathways in corneas of infected M1^-/-^ mice. Following Nanostring analysis, the genes were further analyzed by Metascape and based on this analysis the top two upregulated pathways in infected corneas were: inflammatory response (GO: 0006954) and leucocyte migration (GO: 0050900). The top two downregulated pathways were: erythrocyte homeostasis (GO:0034101) and regulation of DNA-binding transcription factor activity (GO: 0051090); and B and C) Upregulated and downregulated pathways in brain of infected M1^-/-^ mice. The top two upregulated pathways in infected brains were: Hematopoietic cell lineage (GO: ko04640) and positive regulation of vasculature development (GO:1904018). The top two downregulated pathways were: transcriptional regulation by RUNX1(R-MMU-8878171) and muscle structure development (GO:0061061).

We further analyzed these results using Metascape. Upregulated and downregulated genes in infected corneas and brain in each group were analyzed to determine their associations with relevant genetic pathways (Fig 9). The top two upregulated pathways identified in M1^-/-^ mouse corneas were inflammatory response (GO:0006954) and leukocyte migration (GO:0050900) (Fig 9A), which directly correlate with our results. The top two downregulated pathways in M1^-/-^ mouse corneas were erythrocyte homeostasis (GO:0034101) and regulation of DNA-binding transcription factor activity (GO: 0051090) (Fig 9B). The top two upregulated pathways in M1^-/-^ mouse brains were: Hematopoietic cell lineage (ko04640) and positive regulation of vasculature development (GO:1904018) (Fig 9C). Lastly, the top downregulated pathways in M1^-/-^ mouse brains were transcriptional regulation by RUNX1(R-MMU-8878171) and muscle structure development (GO:0061061) (Fig 9D).

Taken together, our results suggest that the loss of M1 macrophages enhances the inflammatory immune response, which is the likely cause of lethality in M1^-/-^ mice.

## Discussion

Herpes simplex virus type 1 (HSV-1), also known as herpes stromal keratitis, is a major cause of impaired vision in humans [4,40] and the type of infiltrating immune cells following acute virus replication and after reactivation from latency may protect from, or cause a pathologic response to, infection [41–44]. After ocular HSV-1 infection and in the absence of previous infection, innate immune cells playing important role in clearing virus from the eye [45–47]. Thus, the influx of cellular infiltrates into the eye have considerable importance after ocular HSV-1 infection [48]. We recently evaluated the time-course of various immune cell infiltrations into the cornea of infected mice from 1 hr to 28 days PI [5]. We noted a significant increase in the total macrophage population after 12 h PI in infected mouse corneas that was primarily due to infiltration of CCR2^+^ migratory macrophages, mostly in M1 status (MHC II^+^). The number of CCR2^-^ resident macrophages, mostly unpolarized (M0), increased gradually over time and peaked at 48 h PI. Further, resident M2 macrophage levels peaked at 12 h PI, concurrent with M1 macrophage infiltration. Thus, macrophages are early-responders to virus infection and their levels correlated strongly with HSV-1 replication as we reported previously [49–52]. In another of our past studies, we showed that macrophages play a very critical role in preventing T cell autoreactivity [53]. Depletion of macrophages by clodronate treatment resulted in CNS demyelination associated with CD4^+^ T cells in ocularly infected mice.

Based on their polarization, macrophages are divided into M1 (classically polarized) and M2 (alternatively polarized) macrophages [7]. Macrophage activation status creates a delicate balance between M1 and M2 phenotypes [8] and increased M2 macrophage expression correlated with higher phagocytosis and higher primary virus replication and latency, while the absence of M2 macrophages did not significantly alter HSV-1 infectivity relative to wild-type mice [14]. To continue our previous work on roles of M1 and M2 macrophages [9,10], we generated knockout mice lacking M2^-/-^ by deleting GATA3 in macrophages [14,54]. Our current study used conditional knockout mice lacking STAT1 expression in macrophages (i.e., lacking M1 expression and referred to as M1^-/-^ mice), to study the role of M1 macrophages in controlling HSV-1 infection, [15,19]. As expected, these mice had significantly less Arg1 than did WT macrophages, while NOS2 levels were similar in M1^-/-^ mice and WT mice. In this study we showed that macrophages from M1^-/-^ mice are more susceptible to infection than are macrophages from WT mice. Previously, human blood monocytes [55–57] and BM-derived macrophages [58–61] were both shown to be resistant to HSV-1 infection. Similar to macrophages isolated from M1^-/-^ mice, macrophages isolated from STAT1^-/-^ mice are also highly susceptible to HSV-1 infection [20]. However, after HSV-1 infection, STAT1^-/-^ macrophages had higher virus titers than M1^-/-^ infected macrophages. Similar to STAT1^-/-^ mice, the absence of responsiveness to IFNα and IFNγ in macrophages isolated from M1^-/-^ mice may contribute to their susceptibility to HSV-1 infection [62–66]. Moreover, studies using an HIV infection model have shown M1 polarized macrophages are susceptible to virus infection and can recruit other cell populations like monocytes and T cells to the inflammation site [67].

Innate immune cells including neutrophils, macrophages, NK cells, dendritic cells and γδT cells play a role in clearing virus from the eye [3], but very few studies have evaluated the role of M1 macrophages and their function in HSV-1 infection. In this study, we demonstrated the significance of M1 macrophages in controlling virus replication in the eyes of mice infected with both virulent HSV-1 strain McKrae and avirulent HSV-1 strain KOS. Similar to this study, STAT1^-/-^ mice had significantly higher virus replication in their eyes than did WT mice [68]. Activation of the IRF/STAT signaling pathway by IFNs and TLR push macrophages toward the M1 phenotype (via STAT1) and IL-13 and IL-4 push macrophages toward the M2 phenotype (via STAT6) [69]. STAT1 and IRF7 are particularly important during virus infections because of their prominent role in regulating antiviral functions. When the activation of these genes is disrupted, successful virus infection can be established [70]. Thus, absence of the M1 phenotype leads to higher virus replication *in vitro* and *in vivo*. Similarly, macrophages polarized toward the M1 phenotype by IFNγ treatment inhibit Ebolavirus (EBOV) infection in mouse and human macrophages and *in vivo* administration of IFNγ reduces the morbidity and mortality rate in infected mice [71]. In contrast to this study, depletion of macrophages by liposomes containing dichloromethylene diphosphonate (L-Cl2MDP) did not significantly alter virus replication in the eyes of infected mice [72]. The lack of any effect on virus replication could be due to the lack of complete depletion of macrophages by L-Cl2MDP that ranges from 70–90% due to the tissues that was tested for the absence of F4/80^+^ expression [73,74].

STAT1 reduces virus infection and inflammatory responses in infected mice [23]. Similar to this study, we have shown that the absence of M1 macrophages in M1^-/-^ mice leads to higher virus replication and mice succumb to infection around day 8–9 PI. This may be due to higher viral replication in M1^-/-^ mice and enhanced inflammatory innate immune responses. STAT1 is one of the transcription factors expressed by M1 macrophages can drive activation of interferon stimulating genes and mediate antiviral activity *in vivo* and *in vitro*, which are crucial for host defense against virus infections [75]. Several other studies have demonstrated the importance of STAT1 in controlling viral replication and associated pathogenesis [64,76]. Absence of STAT1 has a similar effect in mice infected with LCMV, suggesting that STAT1 plays a key role in protection and survival of infected mice [77]. Fully functional STAT1 is crucial to protect the nervous system from neurotropic virus infection and immunopathology [78]. To establish the importance of M1 macrophages in protecting against infection with a virulent strain HSV-1, we performed adoptive transfer of M1 macrophages derived from WT mice into M1^-/-^ mice. The M1^-/-^ recipient mice displayed less virus replication in their eyes and were partially rescued from death after M1 transfer, probably due to the short half-life of macrophages after transfer [79,80]. Hence, our transfer experiment showed that M1 macrophages are crucial in controlling virus replication and thus survival.

In this study we have shown that M1^-/-^ mice are highly susceptible to infection with virulent HSV-1 strain McKrae. Similarly, infection of M1-deficient mice with *Cryptococcus neoformans* increases fungal replication and lung inflammation leading to death of infected mice [19]. In addition to the high susceptibility of STAT1^-/-^ mouse macrophages to HSV-1 infection, they are also highly sensitive to infection by other microbial pathogens and viruses [62–66,78]. In contrast to the higher susceptibility of M1-deficient mice to virulent McKrae virus infection, their infection with avirulent HSV-1 strain KOS caused no death despite the mice having increased corneal scarification. To test the susceptibility of STAT1^-/-^ mice to infection, we infected them with avirulent HSV-1 strain KOS (2 X 10^5^ pfu/eye) without corneal scarification and used WT 129SVE parental mice as controls. All 24 WT 129SVE mice survived ocular infection (100% survival), while 5 of 24 129SVE-STAT1^-/-^ mice survived ocular infection (21% survival). These differences were highly significant using Fisher exact test. Thus, in contrast to the absence of STAT1 in macrophages (i.e., M1^-/-^ mice in this study), the global absence of STAT1 in STAT1^-/-^ mice makes them susceptible to even the avirulent strain of HSV-1. In humans, partial STAT1 deficiency leads to mycobacterial and viral diseases [81,82]. Taken together, our study and studies done in different disease models, illustrates the importance of M1 macrophages in host-pathogen interactions. The absence of STAT1 using LysM-Cre mice may also affect the levels of neutrophils in M1^-/-^ mice but the reduction in the levels of neutrophils in these mice is unlikely to affect HSV-1 infectivity since studies have shown that neutrophils are dispensable for providing protection against HSV-1 infection [83–85].

The process of M1-M2 polarization is highly dynamic and plastic in nature. After induction by pro-inflammatory mediators, M1 macrophages produce significant amounts of pro-inflammatory cytokines (TNFα, IFNγ, IL-6, and IL-12) [12,86]. In contrast, M2 macrophages, which are induced by exposure to IL-4, IL-13, IL-10, or glucocorticoids, do not secrete high levels of pro-inflammatory cytokines but do produce high levels of anti-inflammatory cytokines (IL-10, TGF-β, and IL-1 receptor antagonist), as well as the enzyme, arginase 1 (ARG1) [86,87]. In this study we have shown that macrophages isolated from M1-deficient mice following infection with HSV-1 secreted significantly more GM-CSF, IL-1α, IL-1β, IL-5, IL-6, IL-9, IL-10, IL-12 (p40), IL-12 (p70), LIF, LIX, IP-10, KC, MCP-1, MIP-1α, MIP-1β, MIP-2, MIG, RANTES (CCL5), and TNF-α than did WT infected macrophages. These results confirm the previous report that M2 macrophages participate in the blockade of inflammatory responses [12]. Looking further into the cause of higher viral replication, death, and eye disease in M1^-/-^ mice, we analyzed the mechanism of cellular influx produced after HSV-1 infection into the cornea and brain of infected mice. Using a panel of mouse myeloid innate immune genes, we found that after infection the inflammatory pathway was upregulated in corneas of M1^-/-^ mice relative to WT mice. CCL5, CCL7, IL-1β, CCL12, and CCL19 were all upregulated in corneas of M1^-/-^ mice, which promoted inflammatory reflux into the corneas of infected M1^-/-^ mice. In addition to upregulated CCL5 in corneas of M1^-/-^-infected mice, HSV-1 infected macrophages from M1^-/-^ mice also had significant CCL5 upregulation. CCL5 is chemotactic for T cells, eosinophils, and basophils, and is involved in leukocyte recruitment into inflammatory sites [88]. We also found elevated CCR5 expression in the CNS of HSV-2 infected mice [89] and severe HSV-1 encephalitis was associated with CCR5 upregulation in the CNS of infected mice [90].

We found that increased virus replication and inflammatory cytokines/chemokines in the eyes of mice during primary infection correlated with the absence of M1 macrophages. HSV-1 infection is a severe eye disease and virus infection triggers the host immune response [91]. This influx of pro-inflammatory cytokines and chemokines could produce a cytokine storm that worsens the disease after infection. The term cytokine storm was first used in an influenza infection model to describe the pro-inflammatory nature of cytokine overproduction in a short time and is associated with uncontrolled pro-inflammatory responses and significant immunopathology [92]. Development of a cytokine storm with attendant pulmonary damage has subsequently been reported in other viral, bacterial, and fungal infections and more recently, in COVID-19 [93,94]. Our Luminex and Metascape results clearly demonstrate that the inflammatory pathway takes the lead in pathology of infected M1^-/-^ mice. Pathology associated with HSV-1 infection is a consequence of the host immune response mounted after virus infection [4]. Thus, the absence of M1 macrophages contributes to increased eye disease. However, the absence of M1 macrophages did not alter levels of latency-reactivation in M1^-/-^ mice relative to WT mice. Thus, higher virus replication in the eye and higher eye disease did not correlate with the level of latency-reactivation in the absence of M1 macrophages. The absence of this correlation is consistent with our previous work showing that M2 anti-inflammatory macrophages protect mice from latency-reactivation [9,10,14]. Consequently, M1 macrophages play an important role during primary viral infection.

In summary, our results show that the absence of M1 macrophages was associated with increased inflammation and increased virus replication in the eyes of infected mice. Our study also showed that: 1) the transfer of M1 macrophages can rescue mortality and morbidity in HSV-1 infected M1^-/-^ mice; 2) M1 macrophages play a key role in early phases of infection and in clearing virus replication; and 3) M1 pro-inflammatory macrophages play a more important role than in M2 macrophages during primary HSV-1 infection. Finally, in contrast to our recent published study showing that M2-deficient mice behave similar to WT mice [14], mice lacking M1 macrophages become highly susceptible to HSV-1 infection.

## Materials and methods

### Ethics statement

All animal procedures were performed in strict accordance with the Association for Research in Vision and Ophthalmology Statement for the Use of Animals in Ophthalmic and Vision Research and the NIH *Guide for the Care and Use of Laboratory Animals* (ISBN 0-309-05377-3). Animal research protocols were approved by the Institutional Animal Care and Use Committee of Cedars-Sinai Medical Center (Protocols #5030 and #8837).

### Mice

6-8-week-old C57BL/6 WT mice were purchased from The Jackson Laboratory (Bar Harbor, ME, USA). LysMCreSTAT1flfl [B6.129P2-Lyz2^tm1(cre)Ifo^/J-Stat^tmBiat^] mice of similar age were used with a conditional Stat1 null allele in macrophages and was developed by M. Mueller (Institute of Animal Breeding and Genetics, University of Veterinary Medicine Vienna, Vienna, Austria) [15]. The Cre in LysMCreSTAT1flfl [B6.129P2-Lyz2^tm1(cre)Ifo^/J-Stat^tmBiat^] mice knockout STAT1 in macrophages and Cre is not active in mast cells, NK cells, basophils, and eosinophils and is markedly low in dendritic cells compared with macrophages [95–97]. LysM-Cre is extensively used to achieve genetic manipulation in mouse [98]. The M gene in LysM is expressed weakly in myeloblasts, moderately in immature macrophages, and strongly in both mature macrophages and macrophage-rich tissues [99]. In a comparative analysis of multiple myeloid cell-specific Cre reporter strains, about 90–100% of macrophages were targeted by the LysM-Cre mouse line. Mice with an inactivation of both copies of the LysM gene develop normally and are fertile [100]. The absence of STAT1 expression in macrophages blocked M1 activation in LysMCreSTAT1flfl mice, thus throughout this study we are calling these mice M1^-/-^. 129SVE-STAT1^-/-^ and 129SVE mice were purchased from Taconic Biosciences (Rensselaer, NY, USA).

All mice were bred and maintained in the Cedars-Sinai Medical Center pathogen–free animal facility. Homozygous pups appeared to be healthy and were of normal size and body weight.

### Viruses and cells

Plaque-purified, virulent HSV-1 strain McKrae, avirulent strain KOS, and GFP-VP22 viruses (a gift from Peter O’Hare; Marie Curie Research Institute, Surrey, United Kingdom) were used in this study. GFP-VP22 is a recombinant virus that contains the gene encoding a major tegument protein, VP22, linked to green fluorescent protein (GFP) [101,102]. Rabbit skin (RS) cells were used to prepare virus stocks, culture mouse tear swabs, and determine growth kinetics. RS cells were grown in Eagle’s minimal essential medium supplemented with 5% fetal bovine serum.

### Genotyping by PCR

For genotyping, tail snip samples were collected and lysed at 55°C overnight in 100 μl lysis buffer (100 mM Tris-HCl, pH 8.5, 5 mM EDTA, 0.2% SDS, 200 mM NaCl, and 1 mg/ml proteinase K). After diluting the lysate 1:10 in distilled water, 1 μl was used as a PCR template.

### Ocular infection

Mice were infected with 2 X 10^5^ pfu per eye of McKrae virus as an eye drop in 2 μl of tissue culture media as we described previously [103]. Corneal scarification was not performed prior to infection with McKrae virus. For KOS virus infection, mice received 2 X 10^5^ pfu per eye of KOS virus with corneal scarification as we described previously [104]. Before corneal scarification and ocular infection, mice were anesthetized with ketamine + dexmedetomidine. Following anesthesia and ocular infection, buprenorphine was administered by subcutaneous injection. Buprenorphine was administered again the morning after infection.

### Preparation of macrophages from bone marrow

Femoral bones were dissected and all remaining tissue on the bones was removed. Each bone end was cut off and bone marrow was expelled. Bone marrow cells were cultured for 6 days. To differentiate and activate macrophages, 20ng/ml M-CSF and GM-CSF [Peprotech, Rocky Hill, NJ Catalog no. 315–02 (M-CSF) and 315–03 (GM-CSF)] was added along with the cells to be cultured, as we described previously [14,20]. On day 3, M-CSF and GM-CSF were added again to new media, and cells were returned to the incubator until day 6. On day 6, media was removed, and cells were washed three times with PBS to remove floating cells. Macrophages adhered to tissue culture dishes were scraped off and counted for procedures described below. Flow cytometric analysis were done to determine the percentage of F4/80^+^ (for macrophages) and CD11c^+^ (for DCs) cells in isolated adherent culture following staining the cells with anti-F4/80 APC (Clone BM8; Cat no. 123115) and anti-CD11c BV421 (Clone N418; Cat no. 117330) mAbs from BD Biosciences, San Jose, CA and Biolegend USA. Stained cells were gated for F4/80^+^ and CD11c^+^ cells as shown in S2 Fig. Percentage of positive macrophages detected in this study is in line with previous studies [105,106]. Post experiment data analysis was performed using FlowJo software v10.7.1 (BD Biosciences).

### Activation and infection of bone marrow-derived macrophages

BM-derived macrophages described above were seeded at 2 X 10^5^ cells per well in a 24-well plate. After overnight incubation, medium was replaced with fresh complete DMEM containing 50 ng/ml of murine IFNγ (Peprotech, Rocky Hill, NJ) and 100 ng/ml of lipopolysaccharide (LPS; Sigma-Aldrich, St. Louis, MO) for M1 activation, or complete DMEM containing 10 ng/ml of murine IL-4 (Peprotech) for M2 activation as we described previously [9,10,54]. On the following day, cells were infected with 0.1 pfu/cell or 1 pfu/cell of HSV-1 strain McKrae for 1 hr. Infected cells were then washed three times with PBS and fresh complete DMEM was added to each well. Infected cell monolayers were frozen at 12 and 24 hr PI. After two cycles of freeze thawing of infected cells, virus titer was determined by standard plaque assay using RS cells as described [52].

### Phenotype confirmation of macrophages isolated from M1^-/-^ mice *in vitro*

Monolayers of unpolarized, or M1 or M2 polarized, macrophages derived from WT and M1^-/-^ mice were infected with 1 pfu/cell of McKrae virus at 37°C for 24 hr. Infected cells were harvested and total RNA was isolated as described below to measure NOS2 (M2 marker) and Arg1 (M1 marker).

### Phagocytosis assay

Phagocytosis assay on BM-derived macrophages generated as above from WT and M1^-/-^ mice was performed using a Phagocytosis Assay Kit (IgG-FITC) from Cayman Chemical (Ann Arbor, Michigan) as previously described [14]. Cells were gated for total leukocyte population by SSC-A vs FSC-A. Macrophage (F4/80^+^) population was gated from the total leukocyte population and total F4/80^+^ population was assessed for FITC only which represented percentage of phagocytosis. Phagocytosis was measured using FACS DIVA software.

### Luminex xMAP immunoassay (magnetic bead kit)

BM cells isolated from WT and M1^-/-^ mice were cultured and differentiated into macrophages as described above. Differentiated macrophages were infected with 1 pfu/cell of HSV-1 strain McKrae for 24 h. Media from infected cells were collected and Luminex assays were performed in the Immune Assessment Core at the University of California, Los Angeles (UCLA, CA) using mouse 32-Plex Magnetic Cytokine/Chemokine Kits purchased from EMD Millipore (Billerica, MA) and used according to the manufacturer’s instructions as we described previously [9,22]. Fluorescence was quantified using a Luminex 200 instrument (Luminex Corp, Austin, TX).

### Viral titers from tears of infected mice

Tear films were collected on days 1–7 PI from WT and M1^-/-^ mouse eyes infected with HSV-1 strain McKrae or KOS virus using a Dacron-tipped swab. Each swab was placed in 1 ml of tissue culture medium and squeezed. The amount of virus was determined by standard plaque assay on RS cells as described previously [107].

### Monitoring corneal scarring/corneal disease and angiogenesis

The severity of corneal scarring/corneal disease lesions in mouse corneas was examined by slit lamp biomicroscopy on days 14 and 28 PI. Scoring scale was: 0, normal cornea; 1, mild haze; 2, moderate opacity; 3, severe corneal opacity but iris visible; 4, opaque and corneal ulcer; 5, corneal rupture and necrotizing keratitis, as we described [108]. The severity of angiogenesis on days 14 and 28 PI was recorded using on a 4 pt scale in which a grade of 4 for a given quadrant of the circle represents a centripetal growth of 1.5 mm toward the corneal center. Scores of the four quadrants of the eye were summed to derive the neovessel index (range, 0–16) for each eye at a given time point [108]. Each cornea was examined and the mean ± SEM was calculated for each group.

### *In vitro* explant reactivation assay

WT and M1^-/-^ infected mice were sacrificed on day 28 PI and individual TG were removed and cultured in tissue culture media as described [9,109]. Media aliquots were removed from each culture daily and plated on indicator RS cells to detect reactivated virus. As media from explanted TG cultures was plated daily, we could determine the time at which reactivated virus first appeared in the explanted TG cultures.

### RNA extraction, cDNA synthesis, and TaqMan RT-PCR

TG from individual mice were collected on day 28 PI, immersed in RNA stabilization reagent (RNA Later, Thermo Fisher Scientific, Waltham, MA, USA), and stored at −80°C until processing. Total RNA was extracted as described [110,111]. Levels of LAT RNA from latent TG were determined using custom-made LAT primers and probe as follows: forward primer, 5′-GGGTGGGCTCGTGTTACAG-3′; reverse primer, 5′-GGACGGGTAAGTAACAGAGTCT CTA-3′; and probe, 5′-FAM-ACACCAGCCCGTTCTTT-3′ (amplicon length = 81 bp).

For corneal tissues and cultured cells, total RNA was extracted and 1ug of total RNA was reverse transcribed using a high-capacity cDNA reverse transcription kit (Applied Biosystems, CA) according to the manufacturer’s protocol. mRNA expression levels of genes in the study were determined using: (1) NOS2; assay ID (Thermo Fisher), Mm00440502_m1; amplicon size, 66 bp; (2) ARG1; assay ID, Mm00475988_m1; 65 bp; and (3) gB-specific primers were used to measure viral transcripts in corneas of infected mice on day 4 PI (forward, 5′-AACGCGACGCA CATCAAG-3′; reverse, 5′-CTGGTACGCGATCAGAAAGC-3′) and probe (5′-6-carboxyfluorescein: FAM-CAGCCGCAGTACTACC-3′); amplicon size 72 bp. GAPDH was used as a loading control in all experiments. Quantitative real-time PCR (qRT-PCR) was performed using the TaqMan gene expression assay kit in 384-well plates on ABI QuantStudio 5 (Applied Biosystems, Foster City, CA). Real-time PCR was performed in triplicate for each tissue sample. The threshold cycle (C_t_) values, representing the PCR cycle at which there is a noticeable increase in reporter fluorescence above baseline, were determined using SDS 2.2 software. Copy number of LAT RNA and gB RNA were calculated using a standard curve generated using pGEM5317-LAT and pAc-gB1 DNA, respectively as we described previously [110,112].

### Nanostring gene expression analysis

WT and M1^-/-^ mice were ocularly infected with 2 X 10^5^ pfu/eye of HSV-1 strain McKrae. On day 4 PI, cornea and brain tissues from infected mice were excised and total RNA was isolated. Total RNA concentration of each well remained at 20 ng/μl. Hybridization was performed in a total volume of 18 μl hybridization cocktail (3 μl of reporter Codeset, 2 μl of Reporter plus, 5 μl of hybridization buffer, 3 μl of capture mix, and 5 μl of sample) were mixed and centrifuged. Nanostring analysis was performed in a thermocycler at 65°C for 20 hr. Samples were in a 12-well PCR strip that was loaded into the MAX/FLEX nCounter Prep (Nanostring Tech, Seattle, Washington) loaded with consumables such as reagent plates and a cartridge for the hybridization to take place. The preparation station ran for 3 hr, after which the cartridge was transferred to the Digital Analyzer (Nanostring Tech, Seattle Washington) for imaging analysis. A field of view of 240 was used for the experiment, and the Digital Analyzer ran for an additional 3 hr for each cartridge.

For nanostring gene expression analysis, the gene panel nCounter Mouse Myeloid Innate Immunity Panel was used (Cat no. XT-CSO-MM112-12), which contains probes for 754 genes, including 20 internal reference genes (8 negative, 6 positive, and 6 housekeeping) for data normalization. In addition to the 754 genes used, we customized the panel to include ten more gene probes including three HSV-1 genes (gB, gK, and ICP22) and seven additional inflammatory and lymphocyte T cell markers not included in the original mouse myeloid panel (CD4, CD8, Foxp3, Gzmβ, CD25, CD103 (ITGAE), and Prf1). RNA was purified with RNAeasy (Qiagen), as previously described [110] and analyzed with the nCounter platform. The nSolver software 4.0 was used to analyze nanostring gene expression values and for principal components of probe counts, fold change and pathway analysis (Metascape).

### Statistical analysis

For all statistical tests, p-values less than or equal to 0.05 were considered statistically significant and marked by a single asterisk (*). P-values less than or equal to 0.001 were marked by double asterisks (**). A two-tailed student t-test with unequal variances was used to compare differences between two experimental groups. A one-way ANOVA test was used to compare differences among three or more experimental groups. All experiments were repeated at least three times to ensure accuracy.

## Supporting information

S1 Fig Non-significant differentially expressed macrophage associated genes in wt and M1^-/-^ mice. (PDF)

S2 Fig Analysis of macrophage population in BM-derived culture. (PDF)
