# Supplementary material for: Essential role of M1 macrophages in blocking cytokine storm and pathology associated with murine HSV-1 infection
Source: PLoS Pathog. 2021 Oct 15;17(10):e1009999. doi: 10.1371/journal.ppat.1009999 (PMC8550391; doi:10.1371/journal.ppat.1009999)
Supplement: S1 Fig — (PDF) [file ppat.1009999.s001.PDF]

Non-significant Differentially expressed genes: macrophage associated genes in wt and $M1^{-/-}$ mice

CORNEA

Gene	FOLD	P value
Adgre1	1.22	0.5944
Arg1	1.7	0.1067
CCL17	1	
CCL22	1	
CCL24	1	
CD103 (ITGAE)	1	
CD163	1.25	0.5294
CD25	1.84	0.3894
CD4	1.01	0.9686
CD8	1	
CD80	2.71	0.1713
CxCL10	30	0.0849
CxCL9	18.96	0.0747
Foxp3	1.01	0.5000
H2-Ab1	1.72	0.2675
IFNγ	1.38	0.3927
IFNγr1	1.84	0.2035
IL12a	1	
IL13	1	
IL4	1	
IL6	8.64	0.0905
Mrc1	1.56	0.1401
Nos2	1	
Prf1	1	
Ros1	1	
TNFα	2.7	0.1016
HSV-1 gK	4.94	0.1362

BRAIN

Gene	FOLD	P value
Adgre1	1.12	0.18774034
Arg1	1.19	0.23798877
CD17	1	
CCL22	1	
CCL24	1	
CD103 (ITGAE)	1	
CD163	1	
CD25	1	
CD4	1.4	0.38390478
CD8	1	
CD80	1	
CD86	1	
CxCL10	2.2	0.28608638
CxCL9	1.1	0.68894845
Foxp3	-1.05	0.7446757
HSV-1 gB	1	
HSV-1 gK	1	
HSV-1 ICP22		
Gzmb	1	
H2-Ab1	1	0.98491913
IFNγ	1	
IFNγr1	-1.12	0.25647771
IL12a	1	
IL13	-1.01	0.84447193
IL1β	1	
IL4	1	
IL6	1	
Mrc1	-1.05	0.87091959
Nos2	1	
Prf1	1	
Ros1	1	
TNFα	1	

S1 Fig
