# Supplementary material for: Essential role of M1 macrophages in blocking cytokine storm and pathology associated with murine HSV-1 infection
Source: PLoS Pathog. 2021 Oct 15;17(10):e1009999. doi: 10.1371/journal.ppat.1009999 (PMC8550391; doi:10.1371/journal.ppat.1009999)
Supplement: S2 Fig — (PDF) [file ppat.1009999.s002.PDF]

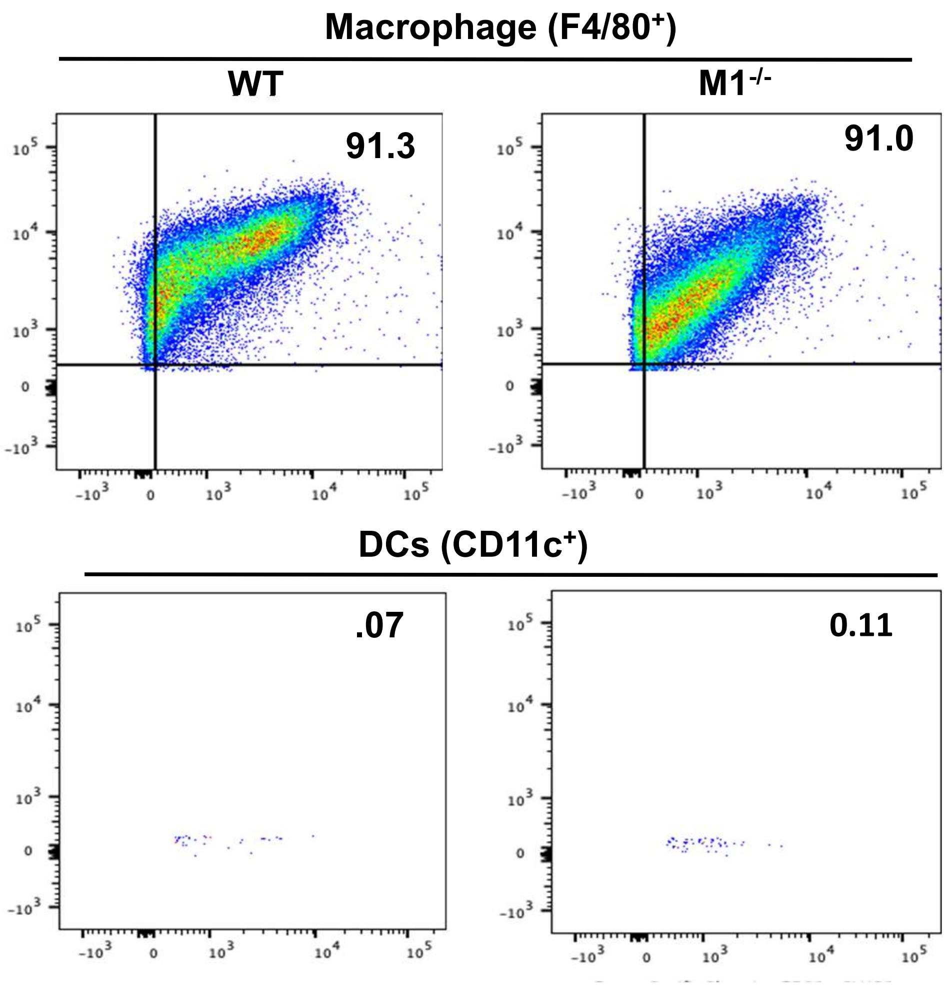

S2 Fig. Analysis of macrophage population in BM-derived culture. After the *in vitro* generation of macrophages, cells from WT and M1$^{-/-}$ groups were harvested, washed and stained with F4/80 APC (for macrophages) and CD11c BV421 (for DCs) antibodies as described in Materials and Methods. The percentage of F4/80^{+} cells in both WT and M1$^{-/-}$ mice were 91.3% and 91%, respectively, while less than 1% of cells were CD11c^{+} in both groups.
